# Engineering of MoSe_2_ and WSe_2_ Monolayers and Heterostructures by DFT-Molecular Dynamics Simulations

**DOI:** 10.1021/acsami.5c07971

**Published:** 2025-06-26

**Authors:** Fabrizio Creazzo

**Affiliations:** Department of Chemistry, University of Zurich, Winterthurerstrasse 190, Zürich CH-8057, Switzerland

**Keywords:** DFT-MD, molybdenum diselenide MoSe_2_, tungsten diselenide WSe_2_, monolayer, heterostructure, transition metal dichalcogenides (TMDs), van der Waals layered material.

## Abstract

Herein, comprehensive modeling and investigation of bulk, monolayers, and heterostructures of 2D transition metal dichalcogenides (TMDs) MoSe_2_ and WSe_2_ have been provided by state-of-the-art spin-polarized density functional theory (DFT) simulations. This work aims to support the rational design of TMD-based (photo)­electrocatalysts for water splitting by incorporating a more realistic description of the catalyst–electrolyte interface. Unlike conventional static or implicit-solvent models, an explicit water environment has been considered at the interface with MoSe_2_ and WSe_2_ monolayers and heterostructures, moving beyond the usual idealized vacuum modeling. Our approach allows for explicit, atomistic interactions at the catalyst–liquid interface at a given temperature, revealing a more realistic modeling and dynamic assessment of interfacial structures. Our simulations reveal that both MoSe_2_ and WSe_2_ exhibit water-repellent behavior, yet preferential hydrogen bonding emerges at specific surface sites. These localized interactions may enhance the catalytic surface activity, underscoring the relevance of capturing interfacial water dynamics in computational models. The study underscores the importance of accounting for explicit liquid water dynamics in DFT-based investigations aiming to engineer monolayer/heterostructure catalytic properties accurately. Here, the key ability to simulate and analyze realistic aqueous environments interacting with semiconducting 2D materials allowed predicting and tuning key interfacial properties, such as electronic structure, water organization, surface electric field, and work function, for the engineering and modeling of enhanced MoSe_2_ and WSe_2_-based interfaces. The lattice parameters, bulk modulus, and electronic structure were also investigated for bulk MoSe_2_ and WSe_2_, which yielded results that are in agreement with the available experimental data. Overall, our study demonstrates that realistic, temperature-dependent simulations of solid–liquid interfaces provide critical insight into the physicochemical behavior of 2D semiconducting catalysts. A similar approach can be applied to other complex facets and interfaces of interest and, hence, possibly help in the design of novel catalysts.

## Graphical abstract

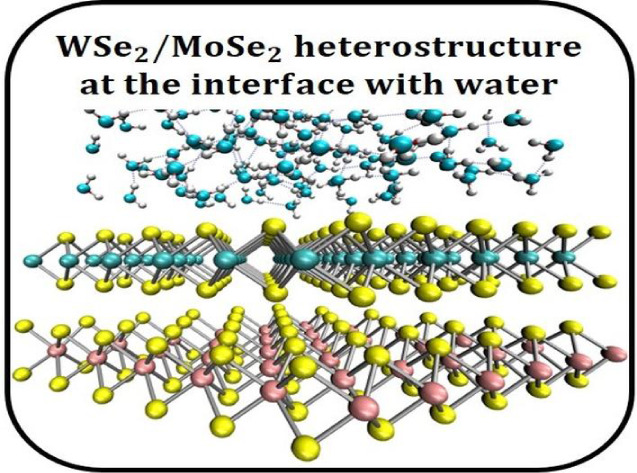

## 1 Introduction

Monolayers of transition metal dichalcogenides (TMDs) are gaining attention as promising materials for a variety of applications, ranging from energy storage,^1^ gas sensing,^2^ and catalysis^3^ to field-effect transistors^4^ and optoelectronic/photonic devices.^5^ TMDs have the chemical formula MX_2_ where M is a transition metal atom (such as tungsten—W or molybdenum—Mo) and X is a chalcogen atom (such as sulfur—S or selenium—Se). The two-dimensional (2D) monolayers of TMDs, such as MoSe_2_ and WSe_2_, have attracted interest due to their improved photocatalytic performance for the hydrogen evolution reaction (HER) in water splitting compared to three-dimensional (3D) TMDs.^6–11^

However, the engineering of a stable and highly efficient photocatalyst for complete solar-driven water splitting continues to be a challenging task.^12^ Specific electronic properties, a broad light absorption range, spatially separated catalytic sites, low overpotentials (for both half-reactions), and interactions with the solvent environment are some key properties of an enhanced photocatalytic process. An intriguing approach is to stack different TMD monolayers (on top of each other) to create 2D heterostructures. It is now widely recognized that layered TMDs can be exfoliated into 2D sheets,^13–15^ much like graphene.

Creating atomically thin 2D-based heterostructures by stacking van der Waals materials such as MoSe_2_ and WSe_2_ opens up a new frontier in condensed matter physics. Their optical band gaps, spanning from the near-infrared to the visible light range, and their strong light-matter interactions make TMD 2D heterostructures suitable for efficient photovoltaic devices.^16^ When two monolayer crystals with matching lattice symmetries are overlaid, a moiré-like superlattice may form due to slight mismatches in their lattice constants or angular alignment.^17^ This phenomenon is seen in systems like WSe_2_/MoSe_2_ heterobilayers, where twist-angle dependence affects moiré excitons.^18^ Since electrons in these atomically thin 2D layers are subject to layer-to-layer coupling, the properties of van der Waals heterostructures are influenced not only by the individual monolayers but also by their interlayer interactions. Recently, various intriguing electrical, optical, and magnetic properties have been observed in these heterostructures. Examples include transport measurements revealing Hofstadter butterfly states,^19,20^ fractional Chern insulators,^21^ gate-tunable Mott insulators,^22^ and unconventional superconductivity.^23^ In addition to advances in electrical transport,^24^ significant progress has also been made in optoelectronic and light-harvesting applications.^25–27^ In particular, MoSe_2_ and WSe_2_ have been engineered and fabricated as 2D heterostructures toward boosting the HER by improving interfacial electron transfer, extending the catalytic surface area, facilitating the exposure of more electrochemically active sites, and tuning specific electronic bands.^7,28–31^

However, directly achieving solar-driven water splitting using a semiconductor–liquid junction in a photoelectrochemical or photocatalytic system requires not only the refinement of semiconductor’s electronic and optical properties but also addressing the subtle interaction between the catalyst surface and the liquid environment at the interface, which is a crucial factor for improving photocatalytic performance.^32^

Although surface science is increasingly focused on identifying key parameters that influence the catalytic activity of MoSe_2_ and WSe_2_ monolayers and MoSe_2_/WSe_2_ heterostructures, the explicit impact of a liquid water environment or solvent on the reconstruction of the surface pattern remains still unattainable. Additionally, the ability of MoSe_2_ and WSe_2_ surfaces to adsorb and dissociate water molecules is still not well-defined at the atomic level. The local molecular reorganization of the aqueous environment near the relevant surface is also largely unknown. This lack of a detailed atomistic understanding of the solid–liquid interface hinders the comprehension of fundamental chemical and physical processes, such as the surface hydroxylation/hydration process, which is a key step for improved performance in photoelectrocatalysts. As highlighted by Bonn and colleagues,^33^ the efficiency of chemical reactions at material–water interfaces is significantly influenced by factors such as the extent to which water can be easily or not easily reorganized. In other words, the structural flexibility or rigidity of the water at the interface plays a key role in the characterization of the electric double layer (EDL). This is essential for charge transfers occurring at the interface and for chemical reactions, such as water splitting. Therefore, it is crucial to thoroughly understand the intrinsic chemical and physical properties of the material–water interface before evaluating its performance as a catalyst.

In previous studies,^32,34–37^ we demonstrated how an explicit bulk water environment at the interface with the catalyst can affect surface reconstruction for improved water-splitting performance. In general, it is known that atoms at the catalyst surface are typically undercoordinated compared to those in the bulk: based on this observation, we can predict many properties of water in the local area at the interface (i.e., interfacial water). The interfacial water usually has a structure and dynamics that differ from those of bulk water. At the solid–water interface, there could be a competition between water–water and water–solid hydrogen bond interactions, leading to a specific orientation of the water dipole moment, which differs from the hydrogen bonding structure found in bulk water.^38^ This competition is influenced by the surface chemistry, particularly the degree of hydrophobicity or hydrophilicity of the surface. The balance between these interactions determines how water molecules interact with the surface and plays a key role in the structural and catalytic behavior of the surface.

However, a significant limitation of most theoretical surface-science studies on MoSe_2_ and WSe_2_ ^7,39–43^ is not only the absence of an explicit bulk water environment (typically water as an implicit model or’only’ a single explicit water monolayer at the best) interacting with the surface catalyst but also the elusive nature of the water interfacial dynamics at a given temperature. The interfacial water dynamics play a vital role in surface reconstruction and the interaction between the material and the water environment. Additionally, the electrostatic attraction between a surface and its surrounding water creates a spatially inhomogeneous charge distribution along the surface normal, generating an electric field. This interfacial electric field is the driving force of the reorganization of water molecules at the interface in a preferential direction.

First-principles molecular dynamics (FPMD) simulations can provide valuable nanoscopic details of the interface, challenging to achieve experimentally (due to nanoscale size and sub-picosecond phenomena), such as the structuring of water and hydration patterns at MoSe_2_ and WSe_2_ monolayers and heterostructures. Herein, by employing state-of-the-art spin-polarized density functional theory with Hubbard *U* correction (DFT-PBE+*U*) molecular dynamics simulations, we provide a comprehensive assessment of (001) MoSe_2_ and WSe_2_ monolayers and MoSe_2_/WSe_2_ heterostructures at the interface with water. The dynamic behavior of interfacial water at room temperature and the interaction/organization of explicit water molecules at the surface catalyst will be investigated to aid in the design of more efficient MoSe_2_ and WSe_2_ monolayer- and heterostructure-based photocatalysts. This approach allows us to explore the interactions at the atomic level, shedding light on the properties and behavior of materials and water in a more realistic condition.

Additionally, the specific structural and electronic properties of bulk, monolayers, and heterostructures of MoSe_2_ and WSe_2_ will be revealed. The (001) surfaces have been identified as active facets that drive the HER and are suitable surfaces for the proper development of 2D heterostructures.^28,30,39,44^

The paper is organized as follows: computational methods that we have tested and adopted are described in Section 2.1. The DFT modeling of bulk MoSe_2_ and WSe_2_ is outlined in Section 3.1; the DFT modeling of MoSe_2_ and WSe_2_ monolayers is in Section 3.2; and the DFT modeling of (001) MoSe_2_/WSe_2_ and (001) WSe_2_/MoSe_2_ heterostructures is in Section 3.3. Water structure at the interface and catalyst–water interactions from DFT-MD are described in Section 3.4, and physical observables such as interfacial electric fields and work functions are discussed in Section 3.5. Conclusions are highlighted in Section 4. Additional analyses and results about convergence tests, the choice of the Hubbard *U* parameter, charge density calculations, DFT-MD simulations with a single water layer at the interface, and DFT-MD at 350 and 400 K can be found in the Supporting Information.

## 2 Methods and Materials

### 2.1 Computational Methods

A series of spin-polarized DFT calculations have been performed on the bulk crystalline structure of MoSe_2_ and WSe_2_, (001) MoSe_2_ and (001) WSe_2_ monolayers, and (001) MoSe_2_/WSe_2_ and (001) WSe_2_/MoSe_2_ heterostructures. DFT-MD with around 50 ps time length has been carried out in the Born–Oppenheimer approach on: (i) (001) MoSe_2_ and (001) WSe_2_ monolayers at the interface with explicit water molecules and (ii) (001) MoSe_2_/WSe_2_ and (001) WSe_2_/MoSe_2_ heterostructures at the interface with explicit water molecules. All calculations and simulations have been performed employing the CP2K program package in the Born–Oppenheimer framework.^45,46^ All of our DFT calculations and simulations were conducted at the Γ point of the Brillouin zone for the electronic representation. This approach requires the use of supercells, that is, several replicas of the unit cell in three-dimensional space (see Figures 1 and 2). Details about the computational modeling of the above-mentioned structures are provided in Sections 3.1, 3.2, and 3.3.

**Figure 1:**
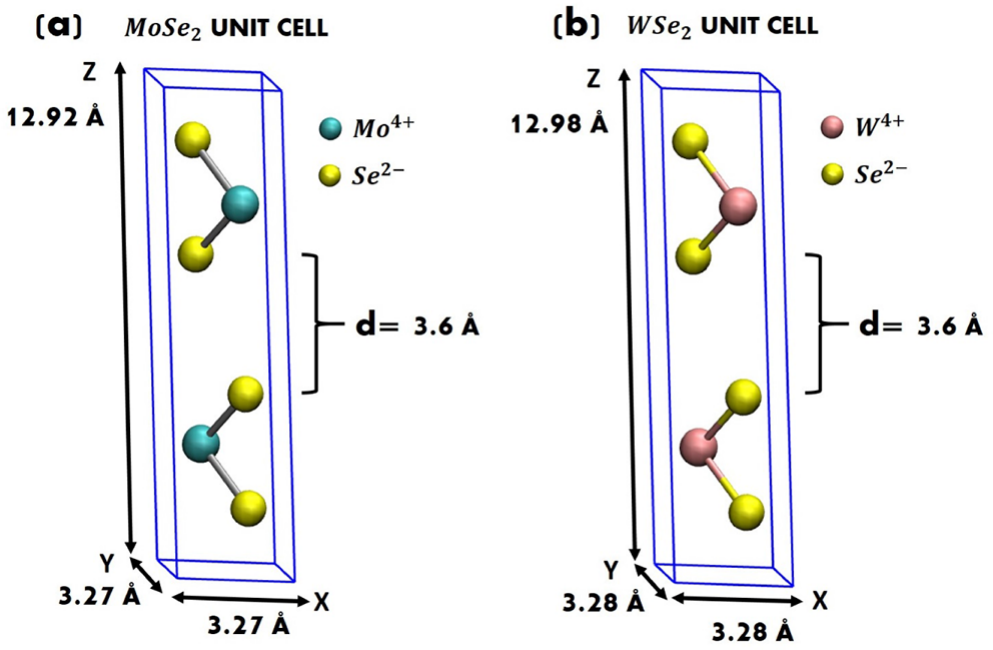
Hexagonal unit cell of (a) MoSe_2_ and (b) WSe_2_ crystalline: 6 atoms in total, 2 Mo^4+^ (W^4+^) and 4 Se^2–^. *d* is the interlayer distance.

**Figure 2:**
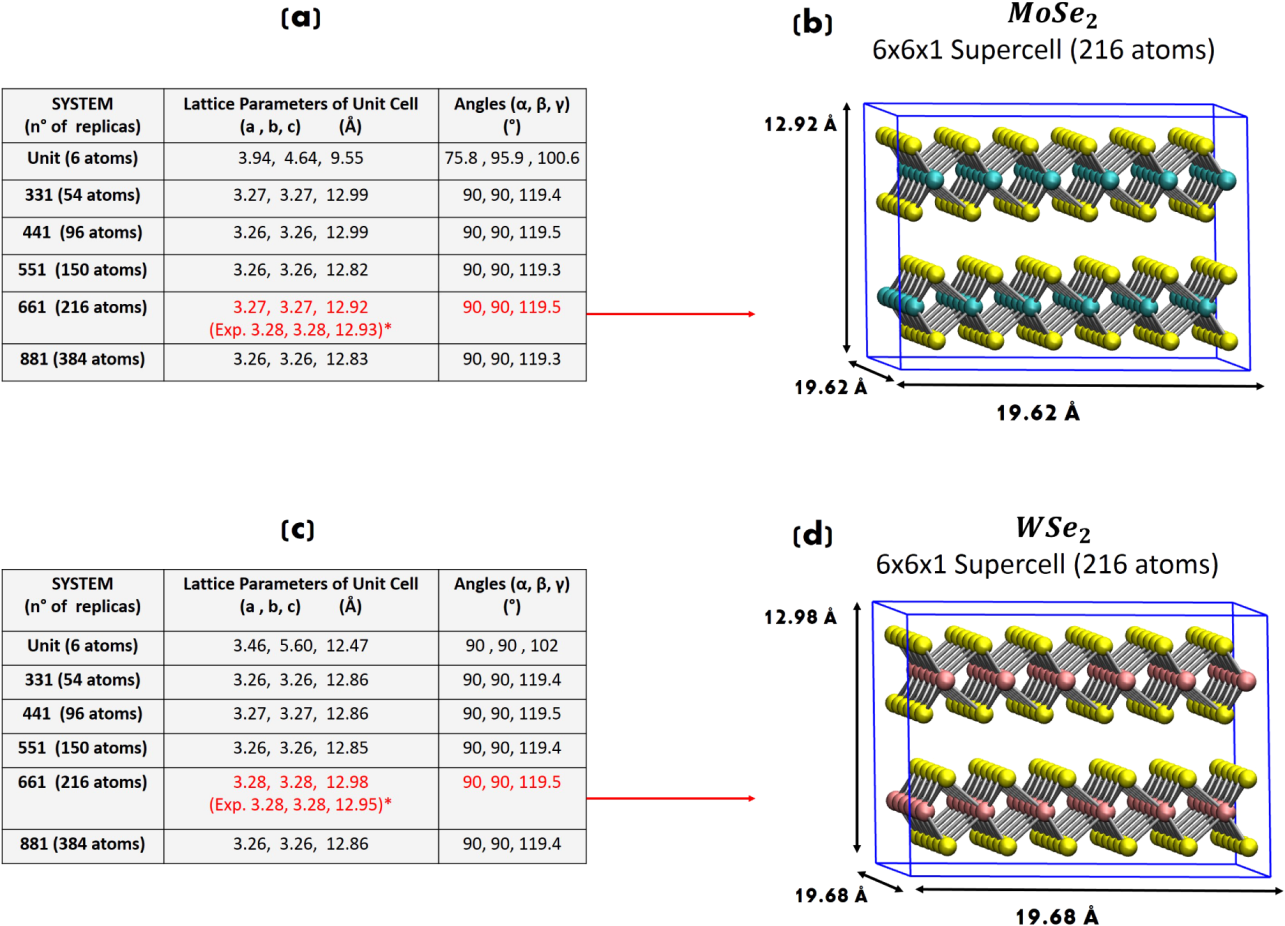
(a) Relaxed lattice parameters and angles for the unit cell and 331, 441, 551, 661, and 881 supercell bulk structures of MoSe_2_. Experimental values from ref. 76. (b) 661 supercell bulk structure of MoSe_2_. (c) Relaxed lattice parameters and angles for the unit cell and 331, 441, 551, 661, and 881 supercell bulk structures of WSe_2_. Experimental values from ref. 77. (d) 661 supercell bulk structure of WSe_2_.

For the exchange-correlation effects, we employed the Perdew–Burke–Ernzerhof (PBE) functional,^47^ which has been demonstrated in previous studies to accurately describe the properties of MoSe_2_ and WSe_2_,^37,48–51^ as well as liquid water^52,53^ and interactions between them. Grimme’s D3 correction^54,55^ for dispersion interactions and Goedecker–Teter–Hutter (GTH) pseudopotentials^56^ have been adopted. We used the DZVP-MOLOPT-SR basis set^57^ along with a 400 Ry plane wave basis set for all atoms, which provides a reliable balance between computational cost and accuracy. The spin-polarized DFT-MD simulations were conducted in the NVT ensemble throughout 50 ps, with the temperature held constant at 300 K using a Nosé–Hoover chain thermostat.^58^ The Velocity-Verlet algorithm^59^ was employed with a time step of 0.5 fs. Periodic boundary conditions (PBC) were applied in all the given three directions of space. The simulation time length of 50 ps aligns with previous theoretical studies on interfaces^60–63^ and it ensures sufficient convergence of dynamical (and structural) properties investigated in this work. Convergence tests of all DFT-MD trajectories in this work are provided in Figure S1.

The PBE functional was supplemented with the Hubbard *U* parameter^64,65^ to address overdelocalization errors in the exchange and correlation of d- and f-orbitals, which can lead to the underestimation of the band gap.^66^ The Hubbard *U* parameter is recognized as the on-site Coulomb interaction energy,^67^ providing a more accurate representation of electron–electron interactions in these systems. While the Hubbard *U* parameter is not universal and varies according to the ab initio protocol (typically depending on the DFT functional, pseudopotentials, electronic state projection scheme, etc.),^68,69^ optimal *U* values were calculated for MoSe_2_ and WSe_2_. Specifically, several values of *U* have been tested for Mo, W, and Se atoms; see Table S2 for details. Results show that adopting *U* = 5 eV for Mo and W atoms and *U* = 4 eV for Se atoms, both MoSe_2_ and WSe_2_ bulk structures behave as semiconductors with the proper/expected bulk electronic band gap (i.e., around 1.2 eV for WSe_2_ ^70,71^ and MoSe_2_ ^70,72^ ). Additional information about the band gap calculations of MoSe_2_ and WSe_2_ bulk structures can be found in Section 3.1. Details about computational resources can be found in Section S7.

The formation energy (or interaction energy) *E* of (001) MoSe_2_/WSe_2_ and (001) WSe_2_/MoSe_2_ heterostructures has been calculated following eq 1:^39,73^

1 $$E_{f}=\frac{E_{MoSe_{2}/WSe_{2}}-\left(E_{MoSe_{2}}+E_{WSe_{2}}\right)}{S}$$

where $E_{MoSe_{2}/WSe_{2}}$ is the total energy of the optimized MoSe_2_/WSe_2_ (or WSe_2_/MoSe_2_) heterostructure, $E_{MoSe_{2}}$ and $E_{WSe_{2}}$ are the total energies of isolated (optimized) MoSe_2_ and WSe_2_, respectively, and *S* is the transverse area of the optimized MoSe_2_/WSe_2_ (or WSe_2_/MoSe_2_) heterostructure.

A uniform background charge and Ewald summation for electrostatics preserve the total charge of the simulation box, following standard procedures in DFT-MD simulations. Electric fields *E*(*z*) and electric potential differences Δϕ are derived entirely ab initio from the optimized electronic wave function and nuclear positions. The electric field at atomic positions is produced by the total charge density (electrons + ions). The electron work function for (001) MoSe_2_ and WSe_2_ monolayers and (001) MoSe_2_/WSe_2_, (001) WSe_2_/MoSe_2_ heterostructures, whether in contact with vacuum or liquid water, has been calculated by integrating the electric field profile as described in refs. 34 and 74. The work function value is estimated as the difference between the electric potential in the vacuum and at the Fermi level. See Section 3.5 for details.

## 3 Results and Discussions

### 3.1 DFT Modeling of Bulk MoSe_2_ and WSe_2_

The stable 2H-phase of MoSe_2_ and WSe_2_ is structured as a hexagonal unit cell belonging to the *P*63/*mmc* crystal symmetry space group (no. 194 in the International Tables of Crystallography), as shown in Figure 1a,b. The unit cell of MoSe_2_ (WSe_2_) is identified by 2 Mo^4+^ (W^4+^) and 4 Se^2–^, for a total of 6 atoms arranged in a lattice of *a* = *b* = 3.27 Å, *c* = 12.92 Å side (*a* = *b* = 3.28 Å, *c* = 12.98 Å for WSe_2_), and α = β = 90°, γ = 119.5°. The bulk coordination geometry is octahedral for Mo^4+^ (W^4+^) and trigonal for Se^2–^. The distance between MoSe_2_ (WSe_2_) layers (i.e., the interlayer distance, *d*) is around 3.6 Å.

Our DFT calculations are based on a Γ-centered grid (Γ point of the Brillouin zone for the electronic representation), excluding the adoption of unit cells as our working models. The modeling of supercells, i.e., a certain number of replicas of the unit cell in 3D space, is needed to explicitly test whether the results have converged. In order to identify the minimum number of replicas (of MoSe_2_/WSe_2_ unit cell) needed to reproduce experimental values in our supercell approach, lattice parameters (and internal atomic coordinates) are therefore geometry-optimized as a function of the simulation box size. Accordingly, full geometry optimizations (atom positions and cell vectors) have been performed on the unit cell and 331, 441, 551, 661, and 881 replica systems of MoSe_2_ (WSe_2_).

The optimizations were initiated on experimental geometries^75–77^ and were carried out without enforcing symmetry constraints. The crystal symmetry of MoSe_2_ (WSe_2_) is preserved by the optimizations. The corresponding relaxed lattice parameters and angles are reported in the tables in Figure 2.

The (661) supercells of MoSe_2_ and WSe_2_ are sufficiently large to converge (within our numerical error) the bulk lattice parameters to *a* = *b* = 3.27 Å, *c* = 12.92 Å and *a* = *b* = 3.28 Å, *c* = 12.98 Å, respectively, with α = β = 90°, γ = 119.5°. These results are in agreement with previous experimental^70,75–79^ and DFT-based^80–82^ studies. The same is valid for relaxed internal coordinates, such as the interatomic distance of around 2.4–2.5 Å between Mo (W) and Se atoms in the (661) bulk supercell structure.

Additionally, the total energy of the aforementioned supercell structures 331, 441, 551, 661, and 881 of MoSe_2_ (WSe_2_) was calculated as a function of volume by fitting the computed values to Murnaghan’s equation of state.^83^ From this, we determined a bulk modulus of 44.8 GPa for the (661) MoSe_2_ supercell. A bulk modulus value of 65.4 GPa was found in our previous paper^37^ for the same (661) WSe_2_ supercell. Our bulk modulus results align with previous experimental and DFT-based values (experimental: 40–50 GPa for MoSe_2_;^84,85^ DFT: 40–50 GPa for MoSe_2_;^86,87^ experimental: 72 GPa for WSe_2_;^88^ DFT: 62.9 GPa for WSe_2_ ^79^), confirming the reliability of adopting the (661) supercell for an accurate estimation of the MoSe_2_ (WSe_2_) bulk properties (lattice parameters and bulk modulus) calculated here

Even if our computational setup is reliable in reproducing the expected structural and mechanical properties (lattice parameters and bulk modulus), the same level of accuracy could not always extend to electronic properties, where discrepancies between experimental and theoretical results can occur due to the approximation of the exchange-correlation energy.

For example, the (electronic) band gap is often significantly underestimated, with errors reaching up to 40%.^66,89^ To enhance the accuracy of theoretically obtained electronic properties, hybrid functionals such as HSE,^90,91^ PBE0,^92^ and B3LYP,^93,94^ as well as screened exchange (sX) hybrid functionals,^95^ have been introduced. Another approach to improve DFT predictions for electronic properties is the use of a’reparatory’ parameter (an on-site Coulomb interaction energy), i.e., the Hubbard term *U* in GGA functionals. In this work, due to a balance between computational cost/demand and reliable accuracy, the PBE functional was supplemented with the Hubbard *U* parameter^64,65^ instead of adopting hybrid functionals. Our previous works on different crystalline structures, such as Co_3_O_4_ ^34^ and TiO_2_,^36^ also highlighted the reliability of the GGA+*U* framework in reproducing electronic properties. It is worth mentioning that there is no consensus on the procedure for determining the optimal value of *U*.^68^ Many previous DFT studies have selected *U* based on various criteria, such as matching structural optimizations to experimental lattice parameters,^96^ aligning the energies of selected Kohn–Sham orbitals with excitation energies measured through’one-electron spectroscopy,’ or adjusting *U* to reproduce the band gap, the energy of states within the band gap, or partially reduced states,^97–99^ as well as the XPS spectrum.^100^ In other cases, *U* has been calculated using a variety of methods.^66,67,101–107^

In this study, the value of *U* for MoSe_2_ (WSe_2_) was specifically calculated so that our DFT-PBE+*U* framework can be useful for calculations focused on catalytic processes. Accordingly, we refer to the band gap energy of the bulk MoSe_2_ (WSe_2_) structure. Experimental reference values for the indirect band gap of bulk MoSe_2_ range from 1.0 to 1.2 eV, obtained by photoelectron spectroscopy,^70,72^ while they range from 0.9 to 1.4 eV^70,71^ for bulk WSe_2_. The computed total density of states (DOS) and projected density of states (PDOS) of (661) MoSe_2_ (WSe_2_) bulk supercells are shown in Figure 3, comparing results from the PBE and PBE+*U* methods.

**Figure 3:**
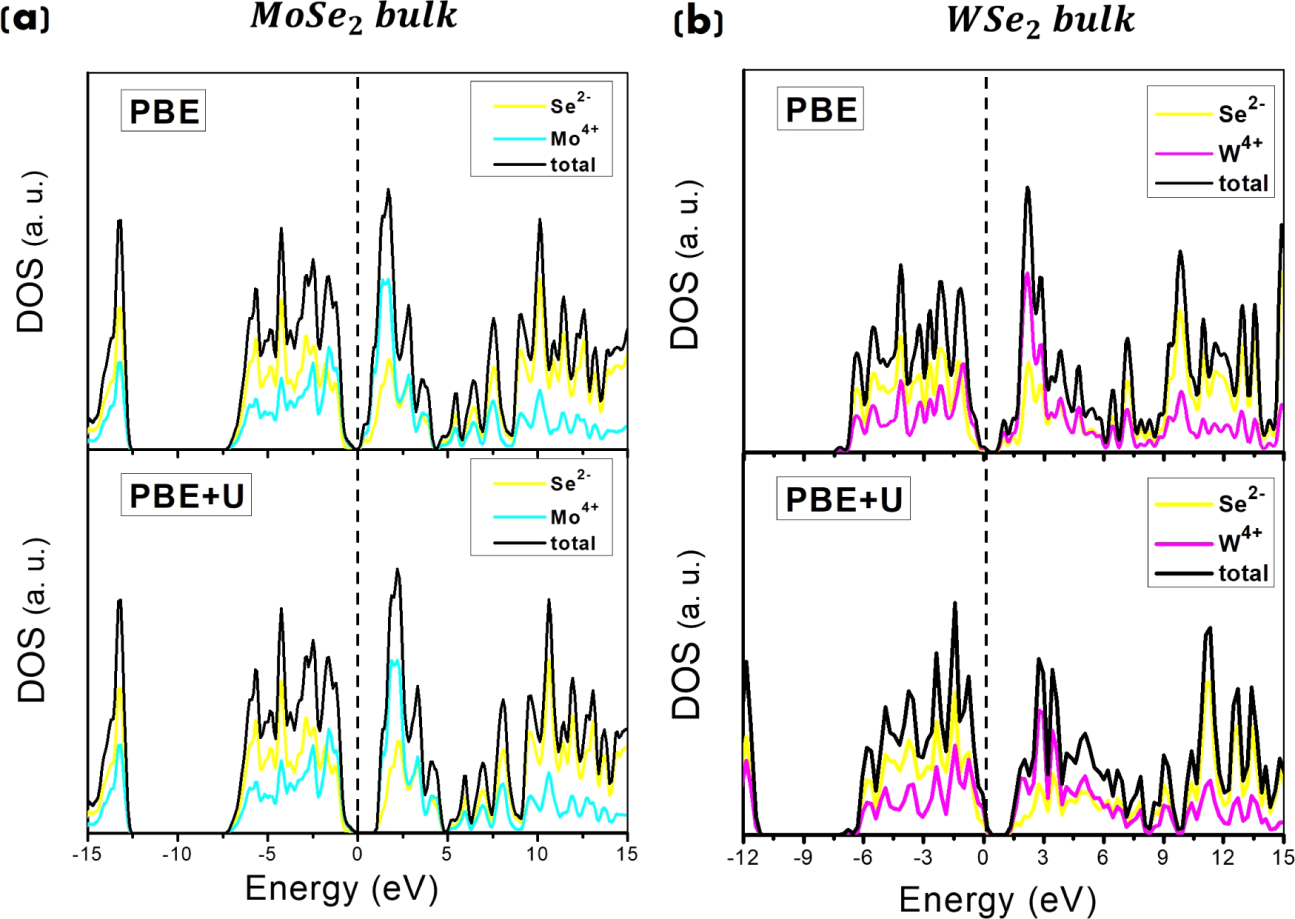
Total DOS and PDOS for the (a) (661) MoSe_2_ supercell and (b) (661) W Se_2_ supercell. PBE calculations on top panels and PBE+*U* (with *U* = 5 eV for Mo^4+^ (W^4+^) and *U* = 4 eV for Se^2–^) on bottom panels, respectively. The Fermi energy level is set to 0.

The comparison confirms the tendency of the PBE functional to underestimate the electronic band gap (see top panels in Figure 3), estimating a value of around 0.2 eV (and 0.7 eV) for bulk MoSe_2_ (WSe_2_).

The PBE functional, when corrected with the Hubbard *U* term, offers enhanced accuracy for predicting energy gaps, bringing computational results closer to experimental data. This correction accounts for strong electron correlation effects in systems with localized *d-* or *f-*electrons, reducing the typical underestimation of the band gap seen with the standard PBE method and improving alignment with experimental values. Our calculations indicate that adopting values of *U* = 5 eV for Mo^4+^ (W^4+^) and *U* = 4 eV for Se^2–^ accurately reproduces the experimental (indirect) band gap energies, yielding values of approximately 1.2 eV for bulk MoSe_2_ (WSe_2_). See bottom panels in Figure 3, in agreement with previously reported experimental data.^70–72,108^

The DOS for bulk MoSe_2_, obtained from the PBE+*U* calculation (bottom panel in Figure 3), reveals distinct electronic contributions: the valence band (VB), with a width of around 7.5 eV, is primarily composed of *Se*-4*p* states, while *Mo*-4*d* states contribute more to the conduction band. The electrical conductivity is therefore given by the free *d*-electrons of the metal ion. This is in agreement with previous computational^87,109^ and experimental^70,72^ studies. Similar conclusions have been found in our previous paper^37^ for bulk WSe_2_ DOS shapes and contributions, having a conduction band *W*-3*d*-derived aligned with results from previous studies.^70,71^

In a nutshell, our findings revealed the (661) supercell of bulk MoSe_2_ (WSe_2_) to be a good compromise between the number of atoms used, dimensions of the box simulation, and reproducing the expected bulk properties (lattice parameters, bulk modulus, electronic states) here calculated. The (661) supercell of bulk MoSe_2_ (WSe_2_) will thus be employed in the next step of investigation, where the bulk structures will be cleaved along the (001) crystallographic plane for the formation of (001) MoSe_2_ and (001) WSe_2_ monolayers, and (001) MoSe_2_/WSe_2_ and (001) WSe_2_/MoSe_2_ heterostructures.

### 3.2 DFT Modeling of MoSe_2_ and WSe_2_ Monolayers

MoSe_2_ and WSe_2_ crystals are van der Waals (vdW) layered materials. It is worth noting that monolayers of TMDs are not composed of a single layer/plane of atoms, as is the case with one-atom-thick materials such as graphene or hexagonal boron nitride (h-BN). Instead, each monolayer is a stable unit formed with hexagonally packed stacking planes, where a plane of Mo (W) atoms is sandwiched between two planes of Se atoms (see Figure 4a,b). These Se–Mo (W)–Se planes are bonded through covalent interactions, exhibiting exceptionally strong intraplane bonding, which ensures structural integrity within each monolayer. Conversely, the interlayer bonding between different Se–Mo (W)–Se monolayers is (relatively) weak, governed by vdW forces, with no chemical bonds connecting the monolayers. This weak interlayer interaction allows for easy separation and stacking of monolayers, preserving their distinct physical and chemical properties.^72,82^

**Figure 4:**
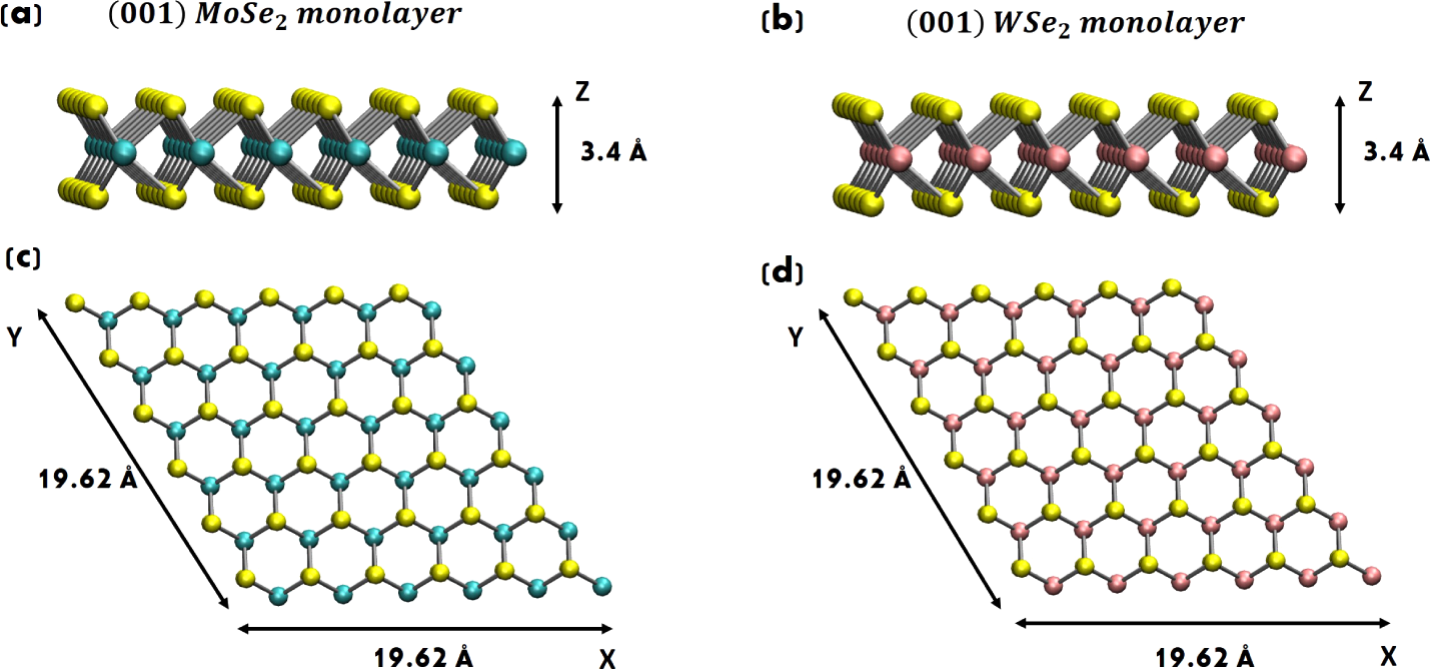
Relaxed (a) (001) MoSe_2_ and (b) (001) WSe_2_ monolayers. 108 atoms in total, where 36 are Mo (W) atoms and 72 are Se atoms. Top view of the (c) (001) MoSe_2_ and (d) (001) WSe_2_ monolayers.

In TMDs, edge planes can be more catalytically active than basal planes, primarily due to their higher density of undercoordinated atoms. However, edge planes are not governed by weak vdW forces but rather by stronger covalent or dangling bond interactions. This makes edge-directed exfoliation or stacking much more difficult, preventing the formation of well-defined 2D heterostructures, of interest in this work, by using edge planes. Previous studies^110,111^ underscore the complexities associated with forming 2D heterostructures using edge planes, highlighting the need for advanced techniques and a deeper understanding of the underlying mechanisms to overcome these challenges. Moreover, the basal planes of TMDs could serve as a more controllable platform for heterostructure formation and tunable catalytic properties, such as doping, strain engineering, defect introduction, and phase transitions, potentially enabling a more realistic and scalable approach to designing improved TMDs.^112,113^

As a result, these crystals can be (easily) cleaved along the vdW gap to obtain Se–Mo (W)–Se monolayers classified as 2D systems, described by anisotropic interactions and anisotropic electronic, physical, and chemical properties,^114^ making them materials of significant interest in various scientific and technological applications. The MoSe_2_ and WSe_2_ monolayers have been modeled in this work, by cleaving the (661) supercell of bulk MoSe_2_ (WSe_2_) along the (001) basal-plane direction, and are shown in Figure 4. From our geometry-optimized structure, we found a monolayer thickness of around 3.4 Å and a Mo (W)–Se bond length of around 2.5 Å.

The variation of the electronic properties of TMDs is usually ascribed to the indirect-to-direct band gap transition when transitions from bulk to monolayer structures are considered (from the 3D to the 2D regime). This indirect-to-direct band gap transition, e.g., at the MoS_2_, has shown great potential and appeal for applications in nanophotonics and photoelectrochemistry.^26,115^ The accurate prediction of the band gap in emerging materials such as (001) MoSe_2_ and (001) WSe_2_ monolayers is therefore essential for advancing the exploration of potential applications. This is particularly crucial for understanding how such materials may behave in various fields such as electronics, optoelectronics, and photonics, where the band gap significantly influences their performance.

Accordingly, PDOS and band gap values have been calculated in this work for (001) MoSe_2_ and (001) WSe_2_ monolayers within our DFT-PBE+*U* setup (*U* = 5 eV for Mo^4+^ (W^4+^) and *U* = 4 eV for Se^2–^). The computed PDOS of the isolated monolayers are shown in Figure 5.

**Figure 5:**
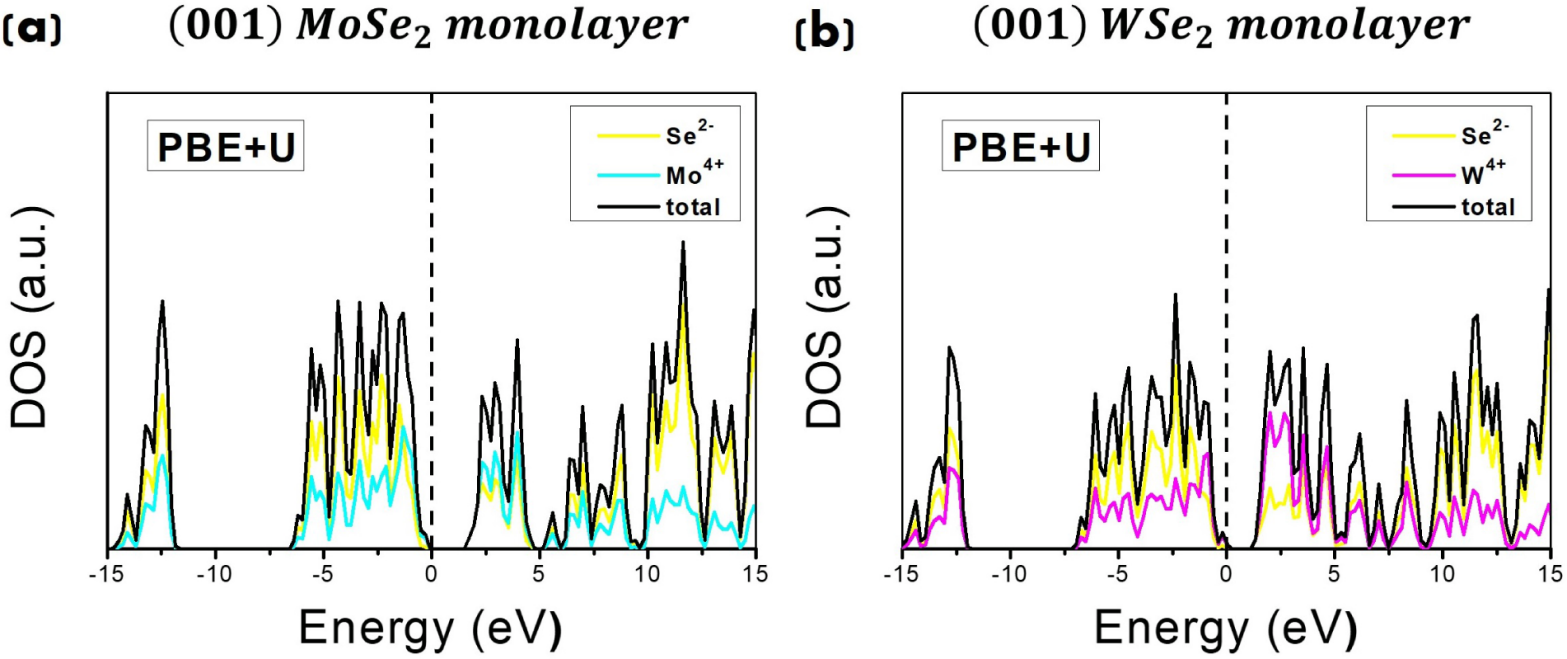
Total DOS and PDOS for (a) (001) MoSe_2_ and (b) (001) WSe_2_ monolayers. PBE+*U* calculations with *U* = 5 eV for Mo^4+^ (W^4+^) and *U* = 4 eV for Se^2–^. The Fermi energy level is set to 0.

Both (001) MoSe_2_ and (001) WSe_2_ monolayers have (direct) band gaps of around 1.6 and 1.7 eV, respectively. Our values are in agreement with previous theoretical data^72,82^ (e.g., 1.57 and 1.67 eV)^82^ and experimental values (1.57 and 1.65 eV)^116^ for (001) MoSe_2_ and (001) WSe_2_ monolayers, respectively.

When transitions from bulk to monolayer structures are considered, MoSe_2_ and WSe_2_ are described as direct band gap materials (not anymore indirect band gap such as in the bulk), and the band gap values of MoSe_2_ and WSe_2_ increase (from indirect 1.2 eV to direct 1.57 eV for MoSe_2_ and 1.65 eV for WSe_2_) in the visible/near-infrared region, all in agreement with previous literature.^39,82,116,117^ Results show that our DFT-PBE+*U* framework is able to describe the aforementioned indirect-to-direct band gap transition, validating our modeling and setup.

### 3.3 DFT Modeling of (001) MoSe_2_/WSe_2_ and (001) WSe_2_/MoSe_2_ Heterostructures

The aforementioned weak vdW interlayer bonding in TMDs, such as in MoSe_2_ and WSe_2_, allows the creation of unique vdW heterostructures by stacking 2D monolayers on top of each other possessing intriguing electronic and optoelectronic properties. Accordingly, we modeled the (001) MoSe_2_/WSe_2_ heterostructure where the (001) WSe_2_ monolayer has been put on top of the (001) MoSe_2_ monolayer (see Figure 6a), and the (001) WSe_2_/MoSe_2_ heterostructure where (*vice versa*) the (001) MoSe_2_ monolayer is on top of the (001) WSe_2_ monolayer (see Figure 6c).

**Figure 6:**
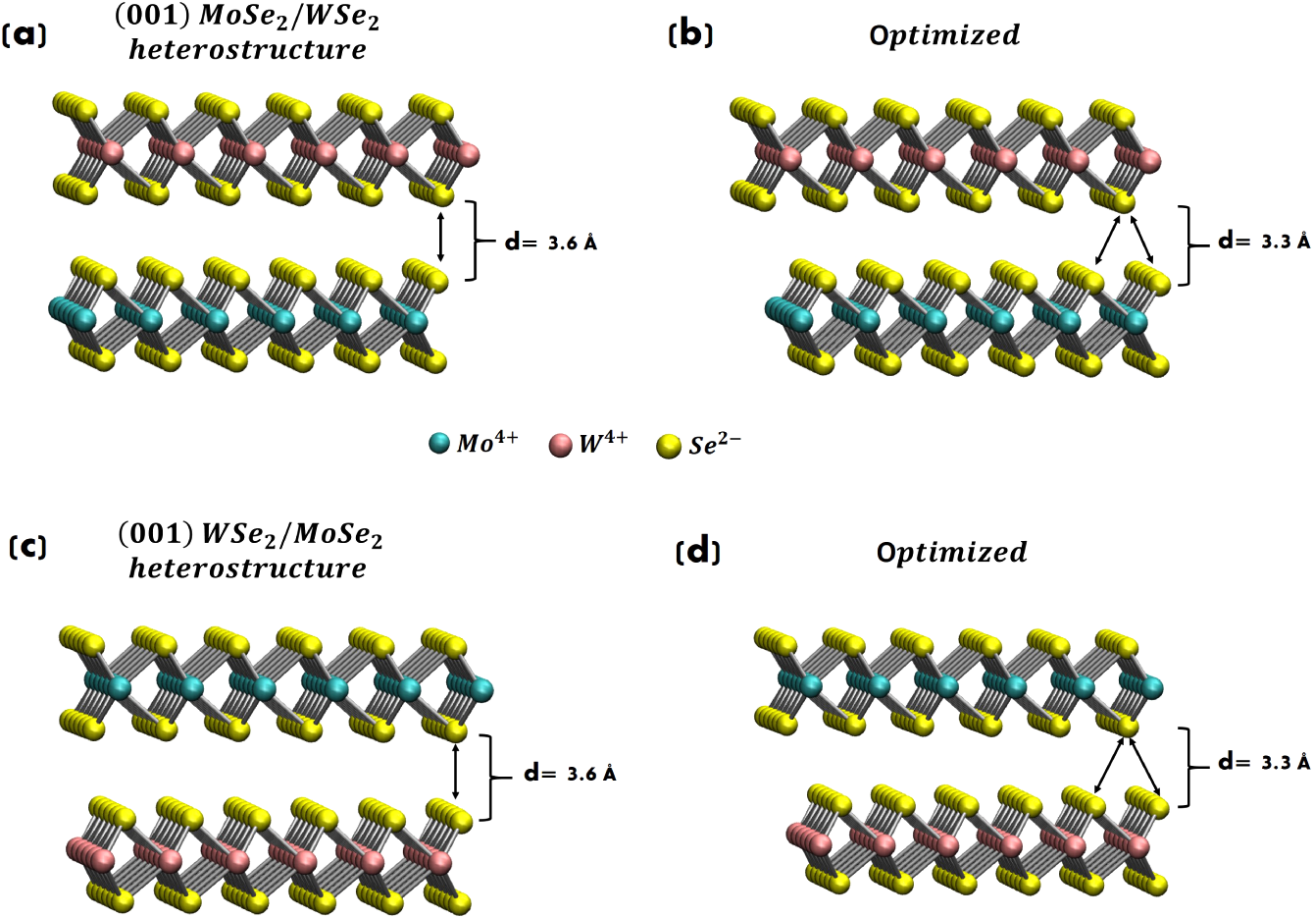
(a) (001) MoSe_2_/WSe_2_ heterostructure. (b) Optimized geometry of (a). (c) (001) WSe_2_/MoSe_2_ heterostructure. (d) Optimized geometry of (c). 216 atoms in total for each heterostructure, where 36 are Mo atoms, 36 are W atoms, and 144 are Se atoms.

The optimized geometries in Figure 6b,d show a (vertical) interlayer distance *d* of around 3.3 Å between monolayers involved in both heterostructures, revealing a typical vdW equilibrium gap and in agreement with previous DFT modeling.^39,118^ Moreover, a (translational) shift of around 1.4 Å of the upper monolayer (with respect to the lower monolayer) occurred during our DFT geometry and cell optimization for both heterostructures, resulting in a lattice mismatch between the upper and lower monolayers. No angle mismatch or ’twist angle’ between such layers has been detected. It is worth noting that not enough lattice constant mismatch or angle rotation has been detected for a proper moiré structure (described by a mathematical moiré factor); however, the slight lattice mismatch could result in the formation of moiré-like superstructures with a large unit cell (on the moiré pattern scale).

In some previous DFT investigations,^73,119,120^ angle mismatches have been considered in heterostructures, sometimes by’manually’ rotating the upper and/or the bottom layer structure. However, in practical devices, the twist angle cannot be controlled with the same precision that is achievable in simulations. Particularly, the twist angle often exhibits spatial nonuniformity,^121–123^ although experimental investigations into the electronic structure of these materials typically rely on devices with a high degree of spatial uniformity (such as in our modeled heterostructures). Accordingly, we consider our optimized structure as heterostructures and not (proper) moiré superlattices.

The formation energies of our heterostructures (relative to isolated monolayers), also called as interaction energy, have been calculated following eq 1, as done in previous investigations.^39,73^ A formation energy value (per unit cell) of around 0.141 ev/A^2^ for both (001) MoSe_2_/WSe_2_ and (001) WSe_2_/MoSe_2_ heterostructures has been estimated. This value falls within the typical range previously observed for vdW materials,^39,73,118^ confirming their thermodynamic stability.

Electronic properties of (001) MoSe_2_/WSe_2_ and (001) WSe_2_/MoSe_2_ heterostructures have been investigated in this work by PDOS and band gap value calculations within our DFT-PBE+*U* setup. The computed PDOS of the heterostructures is shown in Figure 7.

**Figure 7:**
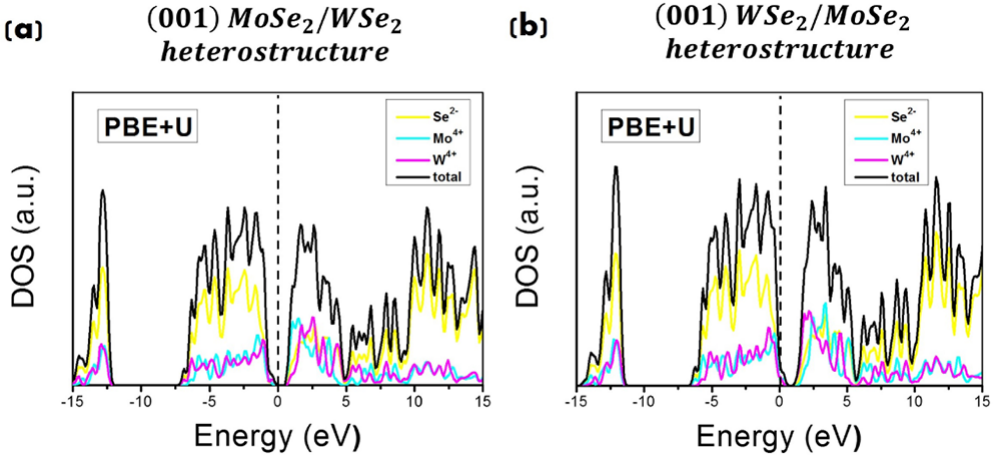
Total DOS and PDOS for (a) (001) MoSe_2_/WSe_2_ and (b) (001) WSe_2_/MoSe_2_ heterostructures. PBE+*U* calculations with *U* = 5 eV for Mo^4+^ (W^4+^) and *U* = 4 eV for Se^2–^. The Fermi energy level is set to 0.

Both systems exhibit indirect band gap values of around 0.55 and 0.45 eV for MoSe_2_/WSe_2_ and WSe_2_/MoSe_2_, respectively, which are smaller than those of the isolated monolayers, aligning well with previous findings for vdW layered materials.^27,39^ See Table 1 for a comparison of the band gap values calculated in this work.

**Table 1:** Calculated Band Gap Values (eV) as a Function of the System Considered, i.e., Bulk , Monolayers, and Heterostructures of MoSe_2_ and WSe_2_

System	Band gap value (eV)
MoSe_2_ bulk	1.2 (indirect)
WSe_2_ bulk	1.2 (indirect)
(001) MoSe_2_ monolayer	1.6 (direct)
(001) WSe_2_ monolayer	1.7 (direct)
(001) MoSe_2_/WSe_2_ heterostructure	0.55 (indirect)
(001) WSe_2_/MoSe_2_ heterostructure	0.45 (indirect)

In these structures, the top of the valence band is predominantly given by the (electronic states of) selenide layers, while the bottom of the conduction band is primarily determined by the (electronic states of) metal layers.

In our modeling, adding a second layer of the same dichalcogenide to a monolayer enhances the indirect band gap, causing a loss of the direct band gap characteristic. As a result, the direct band gap is exclusive to monolayers. However, it is worth mentioning that a few previous theoretical studies on similar heterostructures^124–126^ evidenced that, depending on the interlayer distance, stacking order, the (spatial) direction of the investigations, the kind of dichalcogenide atoms, and mechanical strains applied, it is possible to observe a direct band gap even when considering heterostructures.

As shown in previous experimental and theoretical papers,^124,127–129^ electrons from the basal monolayer (monolayer at the bottom) transfer rapidly (on a femtosecond time scale) toward the upper monolayer (leaving holes in the monolayer at the bottom). This is evidenced in Figure S4a–c, where charge density calculations performed herein on (001) MoSe_2_/WSe_2_ and (001) WSe_2_/MoSe_2_ heterostructures show a higher (electronic) charge density in the upper monolayer.

This charge transfer (from the basal to the upper monolayer) implies a downward shift of the Mo contribution in the conduction band in comparison to the W contribution (see lines in cyan and yellow colors in Figure 7a) at the MoSe_2_/WSe_2_, and a downward shift of the W contribution in comparison to the Mo contribution (see lines in cyan and yellow colors in the conduction band in Figure 7b) at the WSe_2_/MoSe_2_.

### 3.4 DFT-MD on MoSe_2_ and WSe_2_ Monolayers and Heterostructures in Contact with Explicit Water Molecules

Solid–liquid interfaces, such as catalyst–solvent in photoelectrochemical cells, are dynamic environments where atoms and molecules interact with each other over time. Many reactions critical to catalysis, electrochemistry, and materials science occur at these interfaces. DFT-MD allows for the simulation of these processes at a finite temperature, capturing the temporal evolution of both the atomic and electronic structures. The inclusion of temperature-driven dynamics is crucial for realistic modeling of interfaces in real-world conditions, offering a deeper understanding than static calculations. DFT-MD can inherently include the influence of the liquid environment, unlike static models or implicit solvent approaches.

In our previous papers,^32,34–37,130–132^ it has been shown the crucial role of DFT-MD at solid–liquid interfaces by including a full slab of an explicit water environment (closer to the experimental setup) in the context of a better design of (photo)­electrocatalysts for hydrogen production. An explicit full slab of water at the interface, instead of one/few layers of water molecules, has been considered (i) in order to have in our simulation box a proper water environment with a water density of around 1 g/cm^3^, and (ii) because it has been shown in previous investigations^36,131,133–135^ that surface reconstruction (of the catalyst) and chemical reactions which can occur at the interface involve not only one water layer (i.e., the water layer closest to the surface’s catalyst, also called the binding interfacial layer—BIL) but several water layers (also called diffusion layers—DL) above the surface’s catalyst.

The possible surface reconstruction (by adsorbing/dissociating water molecules at the surface), the organization of the interfacial water layers (BIL and DL, which differ from bulk water, e.g., in terms of hydrogen bond arrangement, dipole orientation, etc., largely affecting reactions at the interface), and the complex dynamic behavior at the electric double layer need DFT-MD (instead of static approaches) and an explicit full slab of water for better agreement with experimental findings. As an example, we found that the dynamic hydrogen bond (H-bond) forming/breaking of the water layers at the interface and the specific water organization in the first water layers in contact with the catalyst’s surface can explain the OER rate variation and the OER rate differences at (boron) doped and undoped TiO_2_.^36^

If no water environment or only one explicit water molecule (a water layer at best) is considered at the interface (as done in most of the previous works in the literature^7,39–43^ ), the understanding of the catalyst’s surface reconstruction and the water organization at the interface could be challenging or only partially (sometimes misleading) explained. It is possible to understand that, even if it is experimentally possible to tune the humidity of the interfacial environment (i.e., increasing or decreasing the water ‘quantity’), water is always present at the interface. Then, the theoretical assumption of semiconductors in vacuum models or the adoption of only implicit water (or one explicit water molecule/layer at best) to mimic a liquid environment at the interface could be risky. The explicit structuring of water near hydrophobic or hydrophilic surfaces, as well as the extent and ability of a given surface to reorient water molecules, are critical factors influencing charge transfer and other chemical reactions occurring at the interface.^134,136^

Accordingly, we performed state-of-the-art spin-polarized DFT-MD (50 ps time length) on (001) MoSe_2_ and (001) WSe_2_ monolayers, and on (001) MoSe_2_/WSe_2_ and (001) WSe_2_/MoSe_2_ heterostructures at the interface with 256 explicit water molecules (from a liquid box separately thermally equilibrated with a water density of ∼1.0 g/cm^3^). The simulation boxes and their dimensions for DFT-MD are illustrated in Figure 8. A vacuum layer of 15.0 Å was added above the liquid water along the perpendicular (*z*-direction) to the surface to separate the periodic replicas generated by the applied PBCs, as done in previous works.^34,35,37^ This also hinders the simulation of confined or compressed water, while H-bonding between water molecules prevents them from escaping into the vacuum region.

**Figure 8:**
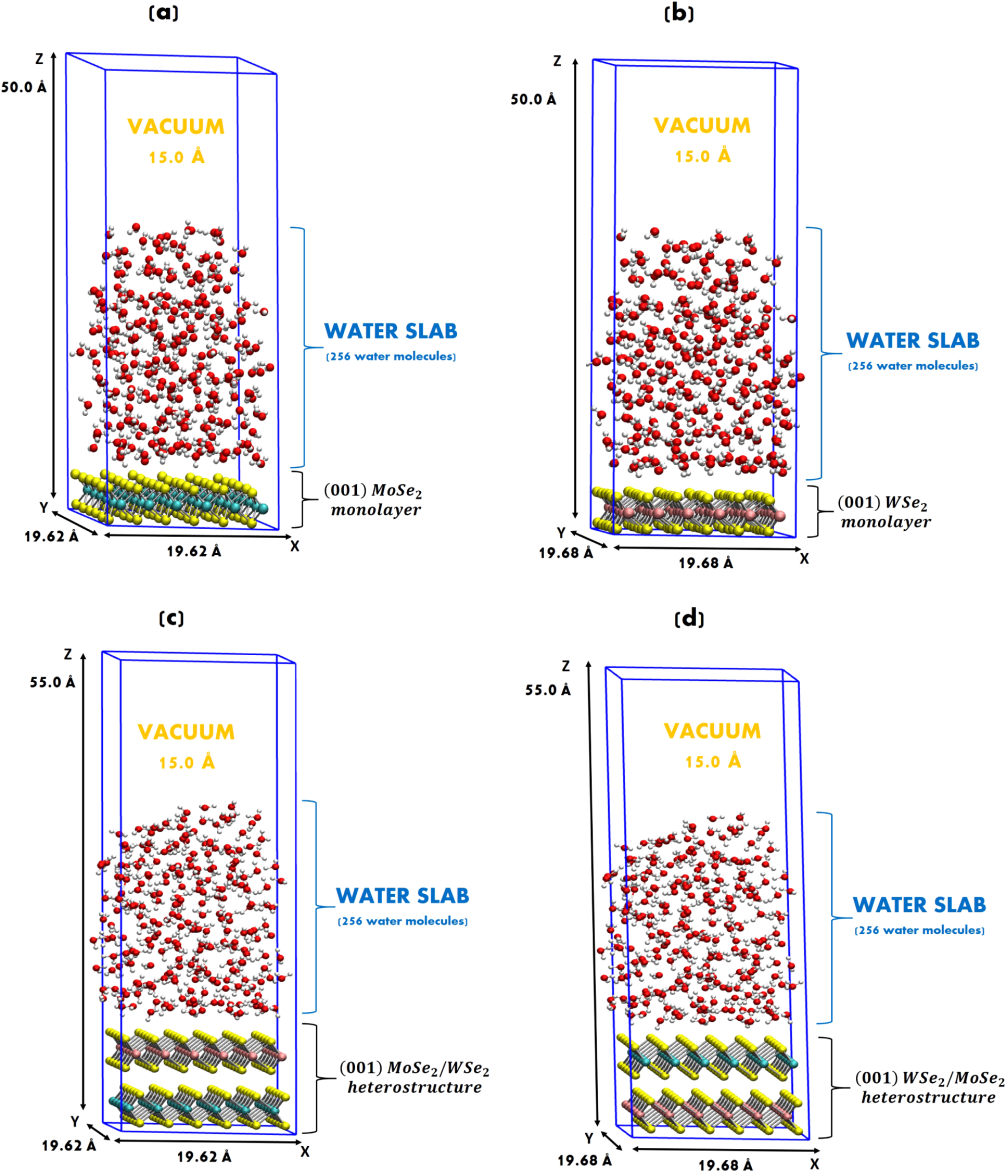
DFT-MD simulation box for (a) (001) MoSe_2_ monolayer and (b) (001) WSe_2_ monolayer at the interface with water. 876 atoms in total for each solid–liquid interface: 108 atoms for the solid (monolayer) and 256 water molecules (=768 atoms). A vacuum of 15.0 Å was added above the liquid water. (c,d) DFT-MD simulation boxes for (001) MoSe_2_/WSe_2_ and (001) WSe_2_/MoSe_2_ heterostructures at the interface with water, respectively. 984 atoms in total for each solid–liquid interface: 216 atoms for the solid and 256 water molecules (=768 atoms). The upper surface is the only one in contact with the explicit water environment. The bottom surface is in contact with the vacuum (15 Å).

Two main descriptors for the catalyst–water interactions have been adopted, i.e., the amount of water molecules that can be adsorbed/dissociated at the surface and the H-bond arrangement of water at the interface. Generally, surfaces in specific directions are characterized by the presence of under-coordinated atoms, and exposing these surfaces to liquid water can lead to the stabilization of them. Water molecules can be either surface-adsorbed in order to ‘passivate’ under-coordinated atoms (by filling electronic states that would otherwise be highly reactive) or can dissociate at the surface (with the hydrogen ion H^+^ binding to one surface site and the OH^–^ hydroxide OH^–^ ion binding to another), leading to the so-called hydration or hydroxylation process of the surface. Under-coordinated atoms stabilized by water adsorption and dissociation often serve as active sites in catalytic reactions. This behavior has been observed in our previous work on specific surfaces such as (110) Co_3_O_4_,^34^ (110) RuO_2_,^35^ (001) WO_3_,^37^ (100) WSe_2_,^37^ and (101) anatase-TiO_2_.^36^

However, herein, during our simulation time of around 50 ps, water molecules are neither adsorbed nor dissociated on (001) MoSe_2_ and (001) WSe_2_ monolayers’ surfaces. The same is valid for (001) MoSe_2_/WSe_2_ and (001) WSe_2_/MoSe_2_ heterostructures. The averaged distance (over the simulation time) between the monolayer’s surfaces and the (first) water layer is of around 3.5–3.6 Å (see Figure 10), resulting therefore in hydrophobic surfaces in terms of the amount of water adsorbed/dissociated on them.^137^ This can be explained by the fact that, contrary to the above-mentioned specific surfaces previously investigated, (001) MoSe_2_ and (001) WSe_2_ monolayers do not expose under-coordinated atoms to be stabilized by water adsorption/dissociation. By the natural atom arrangement and composition of layered TMDs, (001) MoSe_2_ and (001) WSe_2_ monolayers are stable units (Mo^4+^/W^4+^ ions are sandwiched between two planes of Se^2–^ ions) where Se and Mo are not (covalently) under-coordinated. Each Se atom is part of a trigonal prismatic arrangement, where it forms covalent bonds with the Mo (W) atom in the central layer. In this configuration, each Mo (W) atom is bonded to six Se atoms (three above and three below), and each Se atom is bonded to a Mo (W) atom, achieving full coordination. This arrangement results in strong intralayer bonding and weak vdW interactions between the layers. This structural integrity contributes to the stability and unique electronic properties of the MoSe_2_ and WSe_2_ monolayers.

However, in terms of H-bond interactions (and related water dipole moment orientation) between the catalyst’s surface and the water at the interface, we found on average (in our simulation time) that around 55% of water molecules at the interface (in the first water layer closer to the surface) are H-bonded to the (001) MoSe_2_ and (001) WSe_2_ monolayers. The same is also valid for the (001) MoSe_2_/WSe_2_ and (001) WSe_2_/MoSe_2_ heterostructures with an increase to around 70% of H-bonded (interfacial) water molecules. H-bond interactions have also been calculated for DFT-MD simulations performed on the same systems shown in Figure 8 but at 350 and 400 K temperature values. By increasing the temperature, it has been observed a drop by 5–10% (at 350 K) and by 10–15% (at 400 K) of H-bonded water molecules on both monolayers and heterostructures, due to enhanced thermal fluctuations and a more dynamic H-bond interfacial network. Details are provided in Section S6.

The H-bond arrangement found at the interfaces is described by preferential interactions, where the H-bonded water molecules are systematically (H-bond) donors to the (H-bond) acceptor Se atoms at the catalyst’s surfaces, as shown in Figure 9. This is in agreement with the (local) negatively charged Se atoms at the surface that affect the majority of water molecules at the interface to point their positively charged H atoms toward the surface. These preferential H-bond interactions lead to a preferential dipole moment orientation of the water molecules at the interface, which (energetically) prefer to expose (at least one of) their positively charged H^+^ ions toward (pointing to) Se atoms on surfaces.

**Figure 9:**
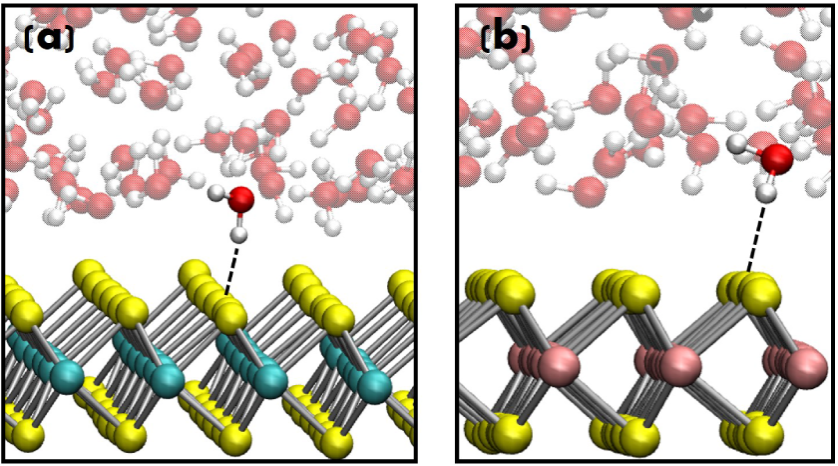
H-bond interactions at the (a) (001) MoSe_2_ and (b) (001) WSe_2_ surfaces in contact with water from DFT-MD. Water molecules are (H-bond) donor to the (H-bond) acceptor Se atoms (in yellow color) at the catalyst’ surfaces.

The radial distribution function in Figure 10 between Se atoms (at the surface) and H^+^ ions (of water molecules in the first layer) reveals an average distance of around 2.5 Å between them. The RDF between Se atoms (at the surface) and O^2–^ ions (of water molecules in the first layer) reveals, instead, an average distance of around 3.5–3.6 Å between them, confirming the preferential dipole moment orientation of water molecules (with the H^+^ closer/pointing to the surface) and H-bond interactions between the catalyst’s surface and the water environment.

**Figure 10:**
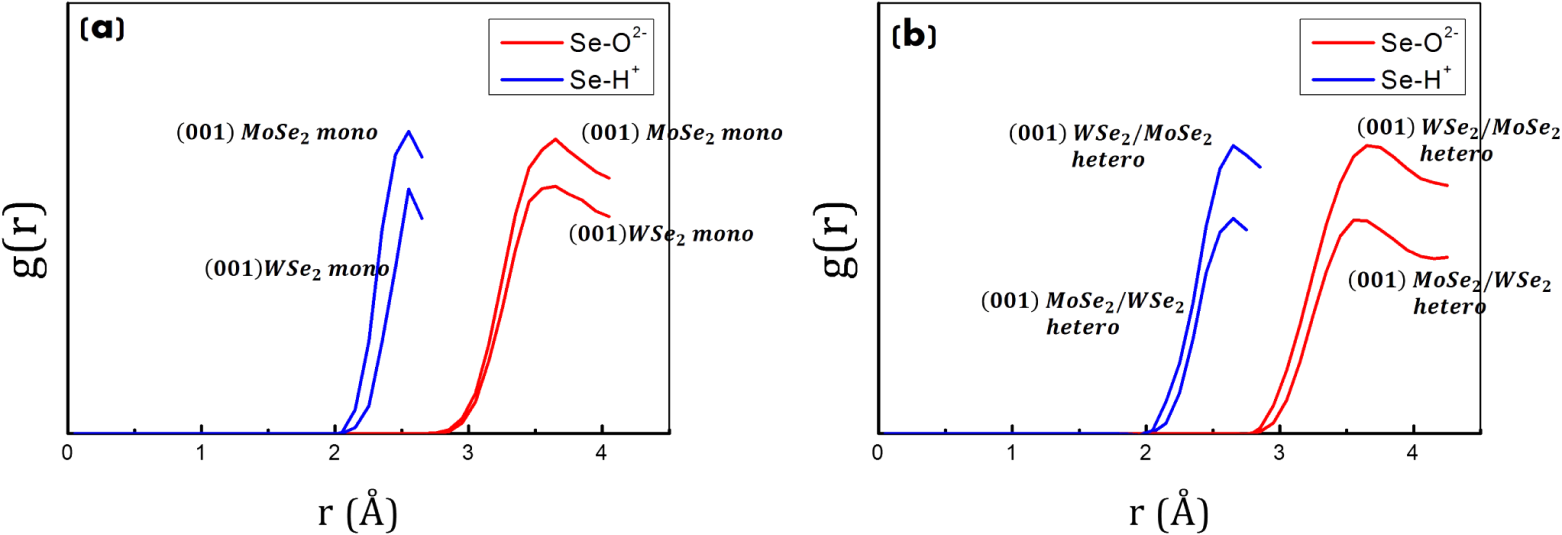
RDFs between Se atoms (at the surface of (001) MoSe_2_ and (001) WSe_2_) and oxygen/hydrogen ions of water molecules in the first water layer. (a) RDFs for (001) MoSe_2_ and (001) WSe_2_ monolayers (at the interface with water). (b) RDFs for (001) MoSe_2_/WSe_2_ and (001) WSe_2_/MoSe_2_ heterostructures (at the interface with water).

For the sake of completeness, water adsorptions/dissociations and H-bond interactions have also been estimated for DFT-MD on (001) MoSe_2_ and (001) WSe_2_ monolayers and heterostructures in contact with only a single layer of explicit water molecules. Details can be found in Section S5. Results show that no adsorption or dissociation of water molecules was observed on the surfaces of monolayers and heterostructures. However, an increased percentage to 95% of water molecules in the single layer make H-bonds with surfaces (instead of 55% and 70% when a full slab of water is considered). This comparison highlights again that the inclusion of a full explicit slab of water at the interface is essential to realistically capture the physicochemical behavior of solid–liquid systems. While a single interfacial water layer may overestimate surface hydrogen bonding due to the lack of competition with water–water interactions, only a multilayer explicit environment can reproduce the complex balance between interfacial and bulk-like hydrogen bonding (see Section S5).

In a nutshell, when considering the amount of water molecules that can be adsorbed/dissociated at the surface, a hydrophobic behavior of both (001) MoSe_2_ and (001) WSe_2_ monolayers and heterostructures is exhibited. However, in terms of H-bond interactions, (001) MoSe_2_ and (001) WSe_2_ monolayers and heterostructures exhibit a much lower degree of hydrophobicity, where the majority of water molecules are H-bonded to the surface in a preferential interaction where the water environment acts as a (H-bond) donor and the catalyst’ surfaces act as a (H-bond) acceptor.

Our theoretical findings are in agreement with previous experimental evidence^138–141^ about the low wettability of TMD surfaces. This water-repellent behavior (low wettability) is beneficial for stability and corrosion resistance in several ways, key parameters for (photo)­electrochemical devices: (i) by minimizing the adhesion and spreading of water droplets, limiting the contact area between the liquid and the material; (ii) by reducing the opportunity for water to penetrate or react with the material, a key factor in preventing corrosion; and (iii) in aggressive environments, such as those with salts or acids, low wettability reduces the deposition and retention of corrosive substances on the surface, enhancing the long-term stability of the material.

At the same time, the preferential H-bond arrangement and dipole moment orientation of the water molecules at the interface (where the majority of the water molecules are H-bond donors to surface ions) could enhance the OER and HER arising, being H^+^ ions’ready’ to be dissociated from water molecules at the interface and surface adsorbed (by negatively charged Se^2–^) when an onset potential will be applied. Studies^36,142,143^ have shown that the alignment of water molecules and their ability to form H-bonds with the catalyst’s surface can significantly affect the activation energy of reactions like water splitting, as well as enhance or inhibit the catalytic activity of materials such as MoSe_2_ and WSe_2_. The aim of the paper is the engineering of chosen TMD monolayers and heterolayers (also together with a water environment) and not to go deeper into water-splitting investigations; however, this could serve as a foundation for further investigations to provide deeper insights into optimizing conditions for the OER and HER, paving the way for improved catalyst design in MoSe_2_ and WSe_2_-based water-splitting applications.

### 3.5 Surface Electric Field and Work Function

Previous investigations, both experimental^127,128^ and theoretical,^124,129^ have already shown charge transfer phenomena in TMD heterostructures, such as those involving MoSe_2_ and WSe_2_. These systems, because of their weak interlayer vdW interactions, are described by electrons and holes localized in different monolayers; usually, electrons from the basal monolayer (monolayer at the bottom) transfer rapidly (on a femtosecond time scale) toward the upper monolayer (leaving holes in the monolayer at the bottom), facilitating the separation of charge carriers between layers. This has been evidenced in Figure S4a–c, where charge density calculations herein performed on (001) MoSe_2_/WSe_2_ and (001) WSe_2_/MoSe_2_ heterostructures show a higher (electronic) charge density in the upper monolayer. See Figure S4a–c for details. This phenomenon is often linked to an interlayer polarization (electric) field, which plays a critical role in electronic and optical properties, such as photoluminescence at reduced energies and tunable properties for applications in optoelectronics and photonics.

Here, instead, with the aim of going beyond the charge transfer between monolayers in MoSe_2_ and WSe_2_ heterostructures, we focus on the electric field at the interface between the catalyst’ surface and the water environment. This electric field, arising from the charge density at the surface, is key for a better comprehension of the subtle interplay at the solid–liquid interface, the structural reorganization of the water at the interface, and to predict catalytic properties.

Surface electric fields have been calculated and compared for: (i) isolated (in-vacuum modeling) (001) MoSe_2_ and (001) WSe_2_ monolayers as well as for (001) MoSe_2_/WSe_2_ and (001) WSe_2_/MoSe_2_ heterostructures and (ii) the same monolayers and heterostructures at the interface with an explicit water environment. The electric field profiles (see Section 2.1 for computational details) have been calculated as a function of the *z*-coordinate perpendicular to the catalyst’s surfaces (see Figure 11). Electric field profiles were computed using configurations derived either from geometry optimizations for isolated bare (in-vacuum) surfaces or from finite-temperature DFT-MD simulations for surfaces in contact with water. For the latter, 50 configurations were extracted at regular time intervals from 50 ps DFT-MD trajectories. For the sake of clarity, only the electric field peaks corresponding to the height of the surface layer (the uppermost layer) are shown in Figure 11.

**Figure 11:**
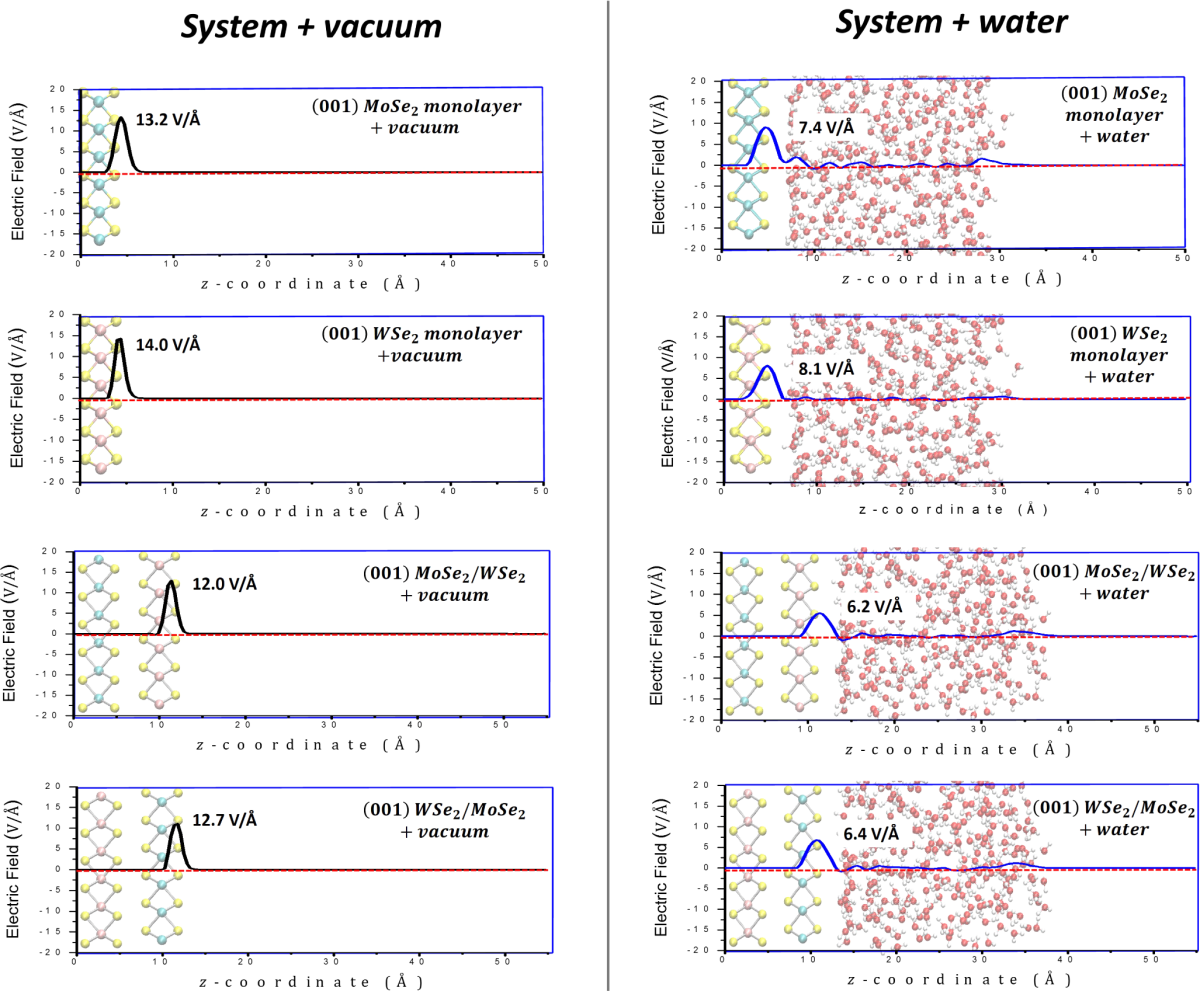
Electric field profiles for (001) MoSe_2_ monolayer, WSe_2_ monolayer, and (001) MoSe_2_/WSe_2_ heterostructure, and (001) WSe_2_/MoSe_2_ heterostructure in contact with a vacuum (panels on the left) or with a water environment (panels on the right). Profiles are plotted along the *z*-direction perpendicular to the surfaces.

Looking at the electric field peaks’ intensities, a systematic decrease of roughly 50% of values occurs from isolated (in-vacuum modeling) monolayers and heterostructures (panels on the left in Figure 11) to monolayers and heterostructures in contact with liquid water (panels on the right in Figure 11). In particular, electric field values of around 13.2 and 14.0 V/Å have been estimated for (001) MoSe_2_ and (001) WSe_2_ isolated monolayers, respectively, whereas ∼7.4 and ∼8.1 V/Å have been estimated for the same monolayers at the interface with the water environment. The same is valid for heterostructures: ∼12.0 and ∼12.7 V/Å for (001) MoSe_2_/WSe_2_ and (001) WSe_2_/MoSe_2_ isolated heterostructures, respectively, whereas ∼6.2 and ∼6.4 V/Å for the same heterostructures at the water boundary. See also charge density calculations in Figures S3 and S4: the results show a consistent trend where the presence of water influences charge localization, in agreement with the electric field decrease shown in Figure 11. Surface electric field values have also been calculated for (001) MoSe_2_ and (001) WSe_2_ monolayers and heterostructures in contact with only a single layer of explicit water molecules, confirming the electric field decreases when explicit water molecules are considered; see Section S5 for details.

The preferential orientation of water molecules near the surface, as shown in Section 3.4, where the majority of the water molecules are H-bond donors (with H^+^ ions pointing toward the catalyst’ surface) to surface ions during DFT-MD, can be explained by the effect of the electric field at the surface catalyst. The electric field at the surface influences water molecules nearby, leading to a dipole alignment of these latter in a preferential direction. This dipole layer partially screens the surface charge (reducing the potential difference between the Fermi level and the vacuum level). The orientation of water molecules near the surface tends to maximize stabilization (with dipoles aligning to complement the charge distribution) by reorienting their dipole moments in that specific direction. Surface electric field affecting the water organization at the interface can also be found in refs. 33 and 144.

By integration of the electric field profiles, it is possible to calculate the work function. Work function values have been calculated for the same systems described above and are shown in Table 2.

**Table 2:** Calculated Surface Work Functions (eV) as a Function of the Interfacial System Considered, i.e., Monolayers or Heterostructures at the Interface with Vacuum or with Liquid Water

System	Work function value (eV)
(001) MoSe_2_ monolayer + vacuum	4.3 (exp. 4.3)^a^
(001) MoSe_2_ monolayer + water	2.4
(001) WSe_2_ monolayer + vacuum	4.6 (exp. 4.4)^b^
(001) WSe_2_ monolayer + water	2.8
(001) MoSe_2_/WSe_2_ + vacuum	3.9 (exp. 3.9)^c^
(001) MoSe_2_/WSe_2_ + water	2.0
(001) WSe_2_/MoSe_2_ + vacuum	4.1
(001) WSe_2_/MoSe_2_ + water	2.1

a Available experimental value from ref. 145.

b Available experimental value from ref. 146.

c Available experimental value from ref. 147.

The systematic decrease of values in peak intensities observed in the electric field profiles upon the introduction of the water environment (above bare surfaces) is directly related to a roughly 50% decrease in the surface work function values for both isolated monolayers and heterostructures when water is at the interface. A similar value decrease has been evidenced in previous works on similar systems^61,145,147–149^ in agreement with our findings. Similar conclusions have also been evidenced in our previous works^34,35,37^ on solid–liquid interfaces.

The presence of an explicit water environment can shift the work function values of an electrode, acting like a doping agent by lowering (and thereby tuning) the energy required to remove an electron from a material’s Fermi level, as defined by the work function. This effect may enhance the surface chemical activity at the interfaces. These surface electric fields could influence surface adsorption, reaction kinetics at the interface, and water-splitting processes. The structure and dynamics of the EDL, along with the chemical processes occurring at the aqueous interface, are essential for accurate modeling of complex catalytic reactions and an understanding of surface interactions, catalytic properties, and electrochemical behavior.

## 4 Conclusions

Herein, state-of-the-art spin-polarized DFT-PBE+*U* molecular dynamics has been employed to shed light on explicit solvent effects on (001) MoSe_2_ and (001) WSe_2_ monolayers and (001) MoSe_2_/WSe_2_, (001) WSe_2_/MoSe_2_ heterostructures trying to go beyond the in-vacuum static modeling. Bulk MoSe_2_ and WSe_2_ have also been investigated in terms of structural, mechanical, and physical properties, such as lattice parameters, bulk modulus, and electronic structures. Understanding the role of water interaction with the surface, especially in terms of water orientation and bond formation, is crucial for designing more efficient catalysts, as it significantly influences surface reactivity and catalytic efficiency. Our findings revealed a transition from indirect to direct and back to indirect band gaps when comparing bulk, monolayers, and heterostructures, respectively. Both MoSe_2_ and WSe_2_ monolayers and heterostructures exhibited hydrophobic character in the presence of water, with no observed water adsorption or dissociation on the catalyst surfaces. However, a preferential hydrogen bonding pattern was identified between the catalyst surfaces and the interfacial water layer, where water molecules act as H-bond donors to the catalyst surfaces.

The precise arrangement of water molecules at the catalyst interface was shown to modulate surface reactivity by influencing the activation energy of chemical reactions.^36,142,143^ In particular, the formation of H-bonds between water molecules and the catalyst surface can enhance or hinder the catalytic activity. The dipole moment alignment of water molecules at the interface can aid in the activation of key bonds, reducing the energy barrier for processes like water splitting, or it may lead to a reduction in catalytic performance if H-bonds interfere with the catalytic process.

Herein, we found that the aforementioned H-bond donor behavior of water molecules at the interface is potentially favorable for accelerating kinetically driven reactions such as the HER. Furthermore, the low wettability of (001) MoSe_2_ and (001) WSe_2_ monolayers and heterostructures we found is beneficial for stability and corrosion resistance, key parameters for (photo)­electrochemical devices. This characteristic enhances the long-term performance of these materials in practical applications.

The surface electric field and corresponding work function values were evaluated to move beyond the already observed interlayer charge transfer between MoSe_2_ and WSe_2_ monolayers, offering deeper insights into how the surface electric field influences the arrangement of interfacial water molecules. A notable 50% reduction in both the electric field and work function values was observed when the monolayers or heterostructures were placed in contact with a water environment. The surface electric field acts as the driving force of the water dipole moments rearrangement, where water molecules at the interface align their dipole moment in a preferential direction in order to screen the surface charge and stabilize the interface environment. Accordingly, the work function value decreases in the presence of water, which can allow easier charge extraction at monolayer and heterostructure water interfaces. These phenomena are critical for promoting enhanced chemical reactions at the interface.

From an experimental standpoint, our findings suggest that

Catalyst Stability: The inherent hydrophobicity/low wettability of (001) MoSe_2_ and WSe_2_ surfaces could imply superior chemical resistance under operational conditions, i.e., enhanced corrosion resistance and structural stability under aqueous conditions, desirable properties for long-term operation in photoelectrochemical cells. This makes them attractive not only as catalysts but also as protective layers in tandem devices;
Surface Engineering: The absence of undercoordinated atoms and the low water adsorption propensity of these surfaces highlight the importance of intentionally introducing defects or dopants to create more catalytic active sites experimentally. Doping the TMDs lattice with nonmetallic (e.g., N, B) or metallic atoms (e.g., Co, Ni, Fe) may induce localized states within the band gap and promote charge localization at specific surface sites. This can modulate the local work function and enhance the interaction with water molecules or reaction intermediates;
Interfacial Tuning: The observed reduction (around 50%) in surface work function upon immersion in water suggests that solvent engineering or external electric field modulation could be viable strategies to tune catalytic activity and enhance charge separation at the interface. Experimentalists can exploit this by tailoring the pH or ionic composition of the electrolyte to tune double-layer effects or by applying gate potentials in electrochemical devices to modulate interfacial charge distribution dynamically;
Layer Stacking and Orientation: Our results confirm fast interlayer charge transfer in (001) MoSe_2_/WSe_2_ heterostructures, supporting the experimental design of layered TMD architectures that favor directional charge transport and efficient carrier separation, key for photocatalysis. Experimentally, deterministic stacking using dry-transfer methods or chemical vapor deposition (CVD) of sequential layers can be employed to fabricate these heterostructures with tailored stacking order and orientation. Our findings suggest that heterostructures with built-in interlayer electric fields may promote charge separation, benefiting photocatalytic processes.

In conclusion, it is important to emphasize that the comprehensive characterization of catalyst–water interfaces achieved in this study would not have been possible by using only a single water molecule/layer or by relying on implicit solvent models, as is commonly employed in many studies. The development of efficient and sustainable heterogeneous catalysts requires an explicit consideration of liquid water (or other solvents) and its dynamic behavior at operating temperatures. This approach can also be extended to other surfaces and interfaces involved in interfacial reactions, aiming to provide an in-depth understanding of these complex systems and phenomena.

## Supplementary Material



## Data Availability

*xyz* coordinates of main structures can be found in the Mendeley data repository (Creazzo, Fabrizio (2025), “xyz main structures”, Mendeley Data, V1, doi: 10.17632/g2xcwt3br4.1, https://data.mendeley.com/datasets/g2xcwt3br4/1). Additional data used only for the publication of this manuscript will be made available on request.

## Notes

Additional analyses and results about convergence test, the choice of the Hubbard *U* parameter, charge density calculations, DFT-MD simulations with a single water layer at the interface, DFT-MD at 350 and 400 K, and computational resources (PDF)
