# Supplementary material for: Engineering of MoSe2 and WSe2 Monolayers and Heterostructures by DFT-Molecular Dynamics Simulations
Source: ACS Appl Mater Interfaces. 2025 Jun 26;17(27):39676–93. doi: 10.1021/acsami.5c07971 (PMC12257458; doi:10.1021/acsami.5c07971)
Supplement: Supplementary file 1 [file am5c07971_si_001.pdf]

SUPPLEMENTARY INFORMATION

Engineering of $MoSe_2$ and WSe_2 Monolayers and Heterostructures by DFT-molecular dynamics simulations

Fabrizio Creazzo[a*]

[a]*Department of Chemistry, University of Zurich, Zurich, Switzerland*

* Electronic mail: fabrizio.creazzo@chem.uzh.ch

S1. CONVERGENCE TEST

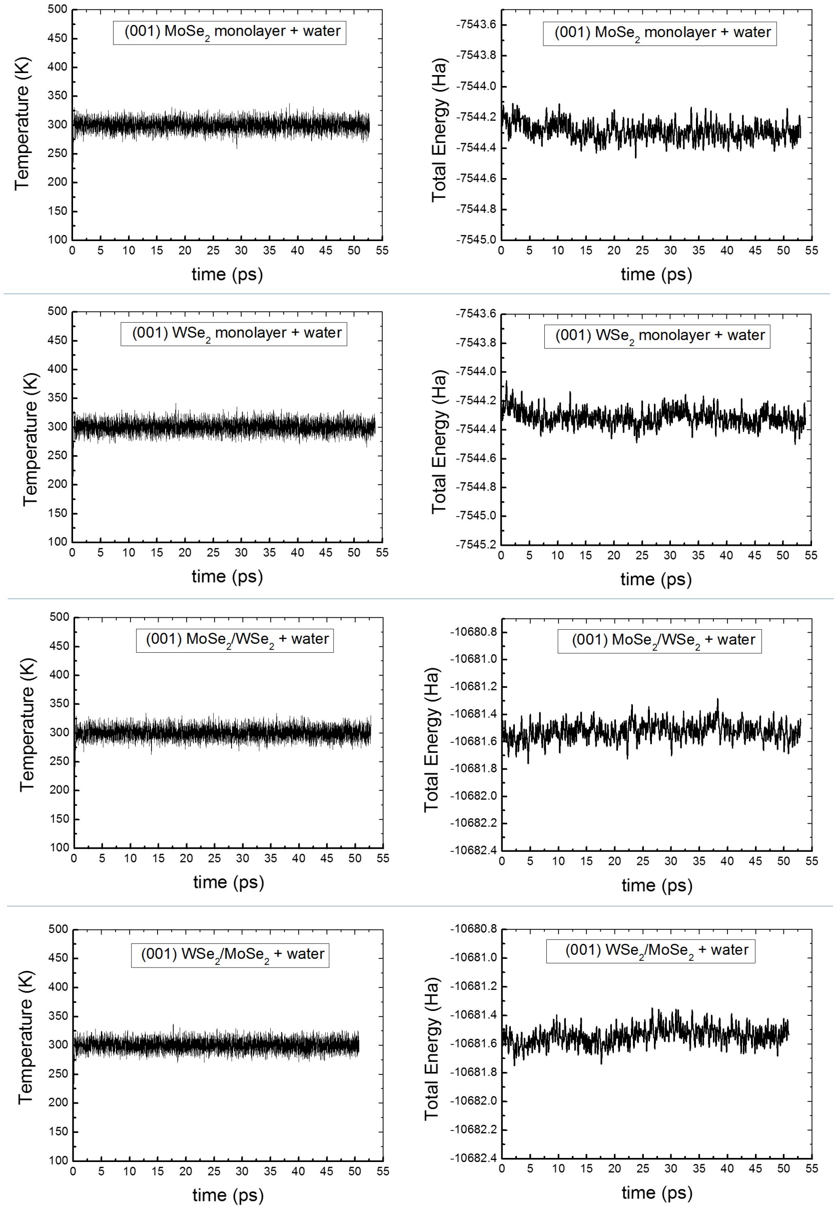

FIG. S1. *Temperature (K) and Total Energy (Ha) as a function of the simulation time for all DFT-MD trajectories. From top to bottom panels: (001) $MoSe_2$ monolayer + water, (001) WSe_2 monolayers + water, (001) $MoSe_2/WSe_2$ heterostructures + water, and (001) $WSe_2/MoSe_2$ heterostructures + water. The NVT canonical ensemble has been adopted where the temperature has been constant at 300 K by a Nosé-Hoover chain thermostat with a time constant of 50 fs. The Velocity-Verlet algorithm has been adopted with a time step of 0.5 fs.*

S2. CHOICE OF THE HUBBARD U PARAMETER

TABLE S2. *Hubbard U values tested for* Mo^{4+}, W^{4+}, *and* Se^{2-} *in* $MoSe_2$ *and* WSe_2 *bulk structures. U values adopted in this manuscript and the (indirect) band-gap value obtained are highlighted in red color. Experimental reference values for the (indirect) band-gap of bulk* $MoSe_2$ *range from 1.0 to 1.2 eV obtained by photoelectron spectroscopy (Ref. 70,72), while from 0.9 to 1.4 eV (Ref. 70,71) for the bulk* WSe_2.

$MoSe_2$ bulk		
**U value Mo^{4+} (eV)**	**U value Se^{2-} (eV)**	**Band-gap value (eV)**
3	3	0.5
4	4	0.9
5	4	1.2
5	5	1.4
6	5	1.7
6	6	1.9

WSe_2 bulk		
**U value W^{4+} (eV)**	**U value Se^{2-} (eV)**	**Band-gap value (eV)**
3	3	0.8
4	4	1.1
5	4	1.2
5	5	1.4
6	5	1.5
6	6	1.7

S3. CHARGE DENSITY ON (001) MOSE$_2$ AND (001) WSE$_2$ MONOLAYERS

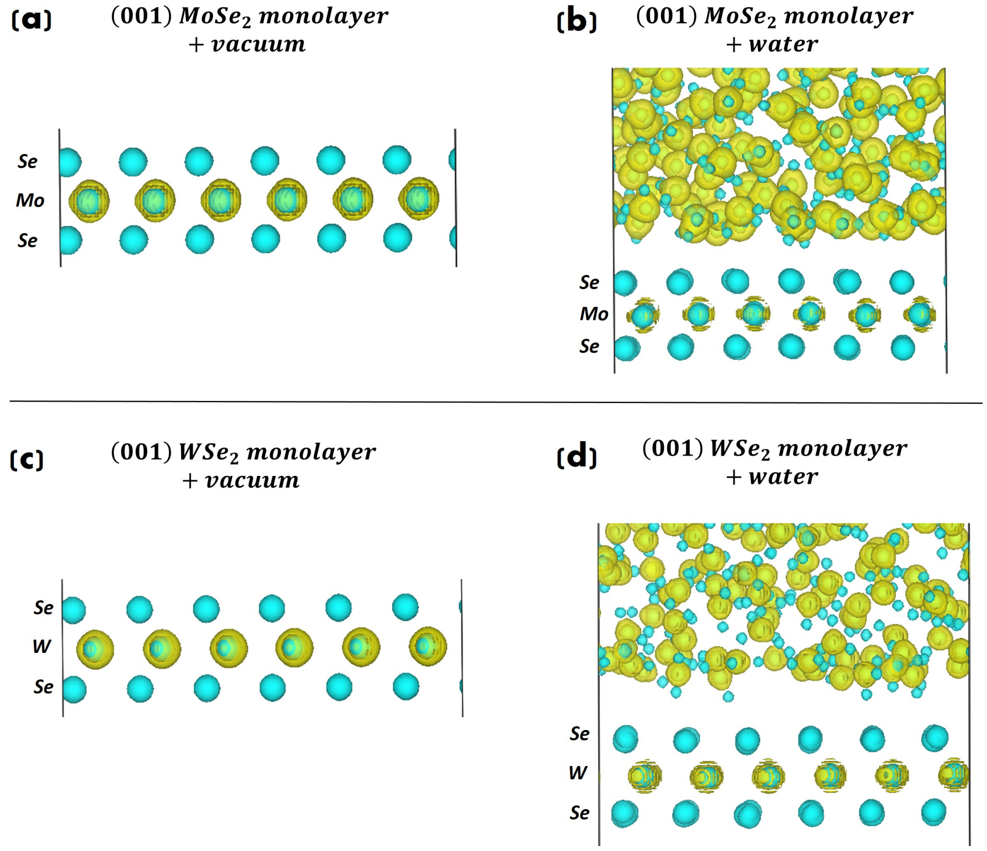

FIG. S3. *Charge density calculations: electronic charge density in yellow color and charge density from nuclei in cyan color. Isosurface value of 0.12* $e^-/Bohr^3$ *has been chosen to emphasize regions of higher electron localization i.e. around metal atoms for (001)* $MoSe_2$ *and (001)* WSe_2*, where it is possible to note differences in the electronic charge density. This choice ensures a clearer differentiation of charge distribution around the metal (Mo, W) atoms compared to the selenium (Se) atoms, filtering out low-density electron clouds (e.g. around Se atoms), and leading to a better interpretation of electronic contributions in the system. This is particularly useful for transition metal dichalcogenides (TMDs) like* $MoSe_2$ *and* WSe_2*, where the metal d-orbitals play a dominant role in electronic properties.*

The charge density isosurfaces strongly localize around the Mo and W atoms, confirming the dominance of metal *d*-orbitals in bonding and electronic interactions. Conversely, Se atoms (Se) exhibit minimal charge accumulation at this isosurface value, filtering out their

low-density electron clouds. This aligns with expectations since Se *p*-orbitals are more delocalized and contribute less to high-density regions.

Charge density calculations on (001) $MoSe_2$ and (001) WSe_2 monolayers show a lower (electronic) charge density around metal atoms when in contact with water (in comparison to vacuum, compare panel *a* with *b*, and *c* with *d*), supporting the surface electric field results shown in Fig. 11 (and accordingly the work function values in Table 2) in the main text.

S4. CHARGE DENSITY ON (001) $MoSe_2$ AND (001) WSe_2 HETEROSTRUCTURES

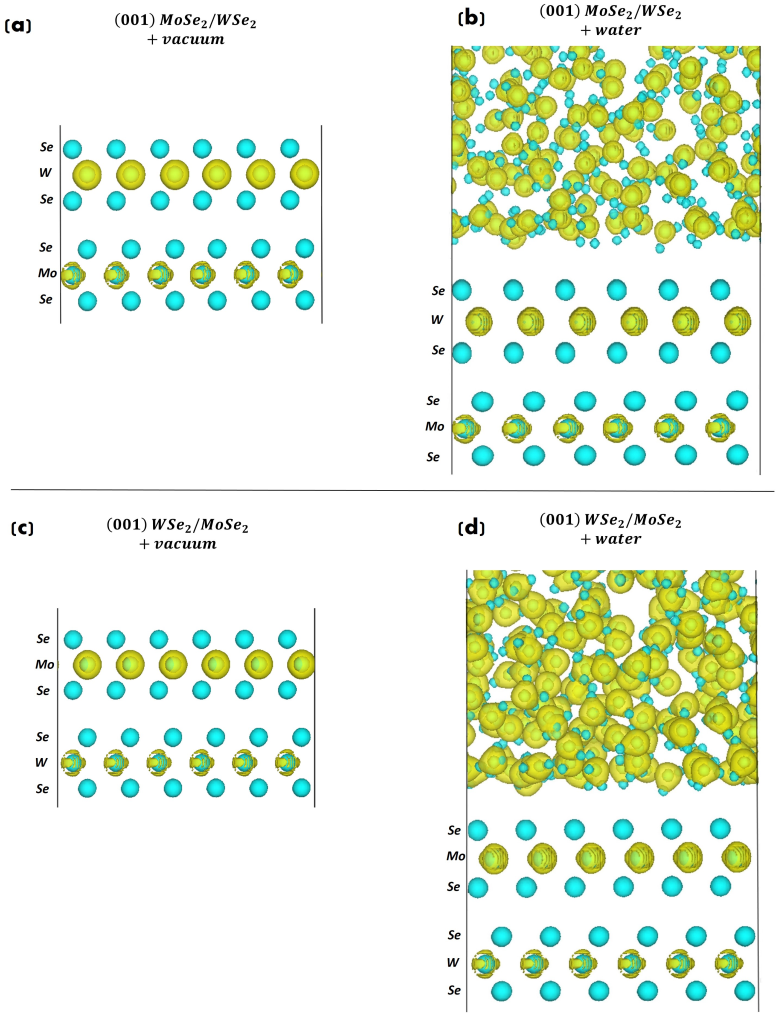

FIG. S4. *Charge density calculations: electronic charge density in yellow color and charge density from nuclei in cyan color. Isosurface value of 0.12* $e^-/Bohr^3$ *has been chosen to emphasize regions of higher electron localization i.e. around metal atoms for (001)* $MoSe_2$ *and (001)* WSe_2*, where it is possible to note differences in the electronic charge density. This choice ensures a clearer differentiation of charge distribution around the metal (Mo, W) atoms compared to the selenium (Se) atoms, filtering out low-density electron clouds (e.g. around Se atoms), and leading to a better interpretation of electronic contributions in the system. This is particularly useful for transition metal dichalcogenides (TMDs) like* $MoSe_2$ *and* WSe_2*, where the metal d-orbitals play a dominant role in electronic properties.*

Charge density calculations on (001) $MoSe_2$ and (001) WSe_2 heterostructures show: 1) a higher (electronic) charge density around metal atoms in the upper monolayer in comparison to the lower monolayer (compare panels *a* and *c*) highlighting the charge transfer (from the basal to the upper monolayer) shown in DOS calculations in Fig. 7 in the main text, and in agreement with previous experimental and theoretical findings [124, 127-129]. See main text for details;

2) a lower (electronic) charge density around metal atoms when in contact with water (compare panel *a* with *b*, and *c* with *d*), supporting the surface electric field results shown in Fig. 11 (and accordingly the work function values in Table 2) in the main text.

S5. DFT-MD ON $MoSe_2$ AND WSe_2 MONOLAYERS AND HETEROSTRUCTURES IN CONTACT WITH ONE EXPLICIT SINGLE LAYER OF WATER MOLECULES

DFT-MD simulations were performed on (001) $MoSe_2$ and (001) WSe_2 monolayers and heterostructures in contact with a single layer of explicit water (28 molecules), as shown in Fig. S5. Simulations were carried out for 25 ps in the NVT ensemble with a 0.5 fs time step. The simulation length is in agreement with the possible water adsorption/dissociation phenomena at the solid surface (and accordingly the structuring of the water at the interface) which typically occur in around 3-5 ps (depending on the system).

During the simulation time, no adsorption or dissociation of water molecules was observed on the surfaces of the (001) $MoSe_2$ and (001) WSe_2 monolayers and heterostructures. The average distance between the monolayer surfaces and the nearest water layer remained consistently in the range of 3.5–3.6 Å, indicating that hydrophobic character with respect to water adsorption or dissociation also found if including a full slab of explicit water molecules at the interface (see main text for details). This behavior has been attributed to the fact that the (001) facets of $MoSe_2$ and WSe_2 do not present undercoordinated surface atoms that would otherwise favor stabilization via water adsorption or dissociation.

However, regarding hydrogen-bonding interactions -and the associated orientation of water dipole moments at the interface- we observed that, on average during the simulation time, approximately 95% of the water molecules in the single interfacial layer form hydrogen bonds with the (001) $MoSe_2$ and (001) WSe_2 monolayers and heterostructures. Instead, when a full slab of explicit water molecules at the interface is considered, it has been found that around 55% and 70% of water molecules at the interface are H-bonded to (001) $MoSe_2$ and (001) WSe_2 monolayers and heterostructures, respectively (see main text for details on the full slab).

This can be rationalized by considering the competition between water–water and water–surface interactions and the limited coordination options per molecule, that is, in the absence of overlying water layers, molecules in the single water layer tend to maximize their

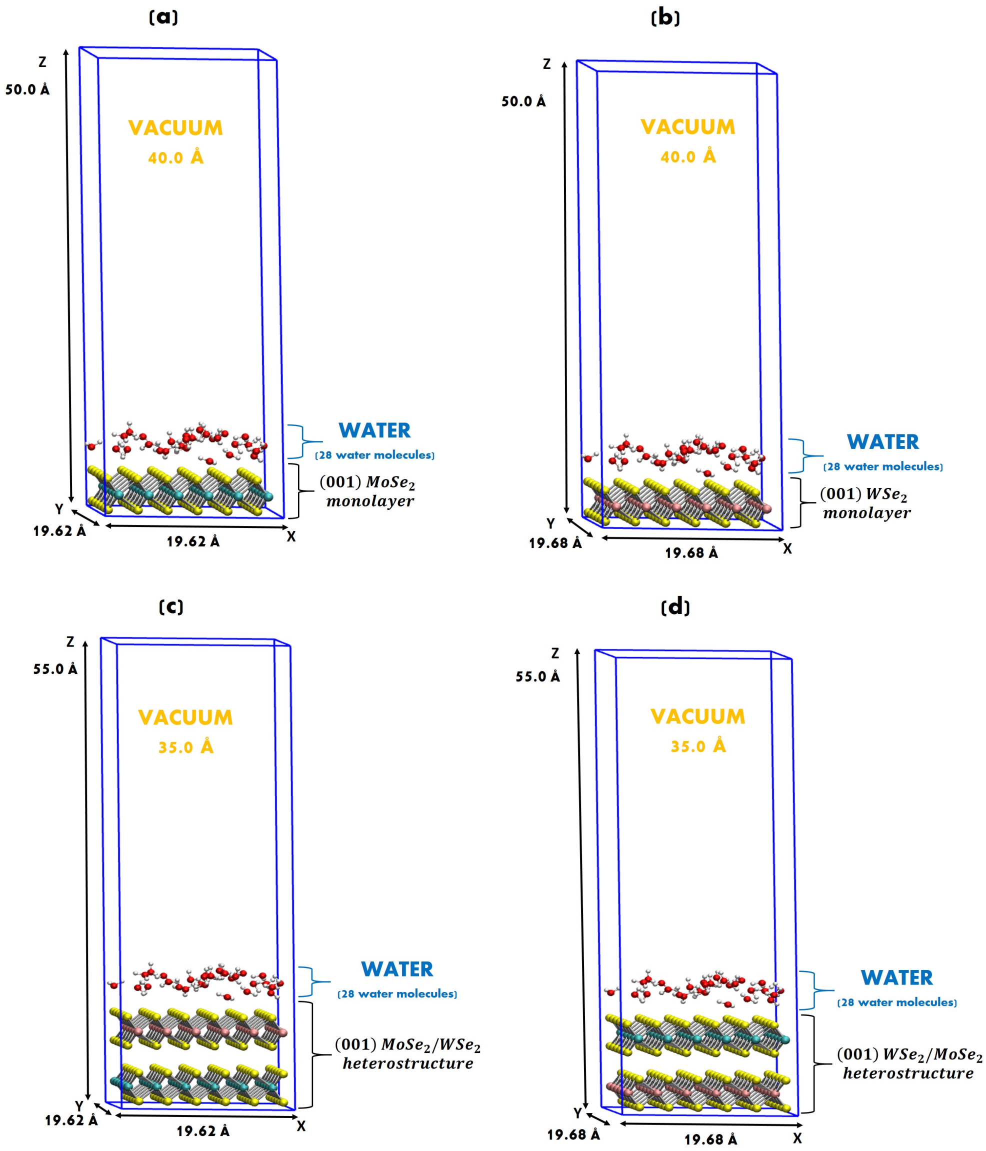

FIG. S5. *DFT-MD simulation box for (a) (001) $MoSe_2$ monolayer and (b) (001) WSe_2 monolayer at the interface with one single water layer. 192 atoms in total for each solid-liquid interface: 108 atoms for the solid (monolayer) and 28 water molecules (=84 atoms). A vacuum of around 40.0 Å was added above the liquid water. (c) and (d) are DFT-MD simulation boxes for (001) $MoSe_2/WSe_2$ and (001)$WSe_2/MoSe_2$ heterostructures at the interface with one single water layer, respectively. 300 atoms in total for each solid-liquid interface: 216 atoms for the solid and 28 water molecules (=84 atoms). The upper surface is the only one in contact with the explicit water environment. The bottom surface is in contact with the vacuum (of around 35 Å).*

interactions with the surface to satisfy their hydrogen bonding capability. This drives nearly all molecules in that single water layer to form H-bonds with the surface, hence the 95% value and the higher hydrophilic-like behaviour of surfaces.

In contrast, in the full water slab, each interfacial water molecule now competes between forming H-bonds with surface atoms and forming H-bonds with neighboring water molecules (both in-plane, and vertically because of the presence of water layers above the interfacial layer). This leads to a reduced percentage (55–70%) of molecules engaging directly in H-bonding with the surface. Interfacial water molecules can now form H-bonds also with neighboring molecules in the water layers above.

These observations underscore again the critical importance of including a full explicit slab of water (bulk-like water density of around 1 g/cm^3) in DFT-based simulations of solid–liquid interfaces. While a single interfacial layer may artificially enhance the extent of hydrogen bonding with the surface due to the absence of competing interactions, only a multilayer water environment could capture a more realistic balance between water–water and water–surface interactions. This balance could governs not only the hydrogen bond network but also the dynamic structuring, dipole orientation, and dielectric response of interfacial water -all of which are central to surface reactivity and electrochemical behavior. Therefore, the adoption of a full explicit water slab is essential to avoid over-simplified or misleading interpretations and to achieve predictive insight into catalytic processes at aqueous interfaces.

Surface electric field values have been also calculated for (001) $MoSe_2$ and (001) WSe_2 monolayers and heterostructures in contact with only one single layer of explicit water molecules. Electric field peaks' intensities (and accordingly the work function values) decrease of roughly 20% in comparison to values calculated from isolated (in-vacuum modeling) monolayers and heterostructure (see panels on the left in Fig. 11 in the main text). This is in agreement with the roughly 50% decrease of electric field peaks' intensities (and work function values) when (001) $MoSe_2$ and (001) WSe_2 monolayers and heterostructures are in contact with a full slab of explicit water (see panels on the right in Fig. 11 in the main text).

S6. DFT-MD AT 350 K AND 400 K ON $MoSe_2$ AND WSe_2 MONOLAYERS AND HETEROSTRUCTURES IN CONTACT WITH WATER

DFT-MD simulations (25 ps time length) on (001) $MoSe_2$ and (001) WSe_2 monolayers and heterostructures at the interface with 256 explicit water molecules have been performed at 350 K and 400 K temperature values. These latter have been chosen being the typical working temperatures of $MoSe_2$ and WSe_2 TMDCs as catalyst/electrode in photocatalysis and electrocatalysis of interest in this paper. The simulation boxes and their dimensions for DFT-MD are the same illustrated in the main text in Fig. 8.

The same descriptors used for DFT-MD at 300 K (in the main text) have been adopted to describe the catalyst-water interactions, i.e. the amount of water molecules that can be adsorbed/dissociated at the surface and the H-bond arrangement of water at the interface. No water adsorption/dissociation phenomena at the catalyst' surfaces have been observed at 350 K and 400 K, confirming the hydrophobic-like behaviour of (001) $MoSe_2$ and (001) WSe_2 surfaces already found at 300 K.

However, the percentage of water molecules forming H-bonds with (001) $MoSe_2$ and (001) WSe_2 monolayers or heterostructures slightly decrease at elevated temperatures. In particular, at 300 K, we observed that around 55% (monolayers) and 70% (heterostructures) of interfacial water molecules form H-bonds with the surface (see main text for details). Instead, these percentages drop by 5–10% at 350 K, and by 10–15% at 400 K on both monolayers and heterostructures. This percentage decreasing can be mainly attributed to the enhanced thermal motion at elevated temperatures, which destabilizes hydrogen bonding by increasing molecular mobility and reducing orientational ordering. In particular, thermal fluctuations increases the kinetic energy of water molecules, making H-bond lifetimes shorter with more frequent H-bond breaking/reforming. Consequently, the dynamic H-bond network at the interface becomes more dynamic and transient, and fewer water molecules are oriented favorably toward the surface to sustain stable H-bonds. These trends emphasize again the importance of explicitly accounting for temperature effects when modeling solid–liquid interfaces and their catalytic behavior.

S7. COMPUTATIONAL RESOURCES

Here below a direct comparison between the computational resources required for explicit, finite-temperature simulations and more conventional static approaches. Calculations have been performed on *Alps*-computer cluster infratructures (see *https://www.cscs.ch/computers/alps* for system specification) of the Swiss National Supercomputing Centre (CSCS).

- DFT-MD with a full slab of explicit water: each of our DFT-MD simulations was performed on (001) $MoSe_2$ and (001) WSe_2 monolayers and heterostructures in contact with a full explicit water slab (256 molecules), totaling 876 to 984 atoms per simulation cell. Simulations were carried out for 50 ps in the NVT ensemble with a 0.5 fs time step.
On average, a single trajectory of 50 ps required:
1000 node-hours (64'000 core hours)

- DFT-MD with a single layer of explicit water: each of our DFT-MD simulations was performed on (001) $MoSe_2$ and (001) WSe_2 monolayers and heterostructures in contact with a single layer of explicit water (28 molecules), totaling 192 to 300 atoms per simulation cell.
On average, a single trajectory of 50 ps (with a 0.5 fs time step) required:
800 node-hours (51'200 core hours)

- DFT (static) geometry optimization with a full slab of explicit water: each of our calculations was performed on (001) $MoSe_2$ and (001) WSe_2 monolayers and heterostructures in contact with a full explicit water slab (256 molecules), totaling 876 to 984 atoms per simulation cell.
On average, for each calculation, it is required:
200 node-hours (12'800 core hours)

These values clearly indicate that the DFT-MD simulations with a full slab of an explicit water environment (256 molecules in our case) are five times more demanding than static calculations (1000 *vs.* 200 node-hours), and around 25% more expensive than DFT-MD

simulations using a simplified single-layer (explicit) water model (1000 *vs.* 800 node-hours). Despite this cost, the added computational effort provides critical physical insights that static (and e.g. implicit solvent) models cannot deliver. In particular, our DFT-MD framework captures i) hydrogen-bond dynamics and orientation of interfacial water molecules, ii) formation of electric double layers and associated field effects, iii) time-averaged structural fluctuations and water layering, essential for evaluating real interfacial stability, iv) dynamic screening and work function modulation by solvent polarization. These effects are crucial when modeling catalyst–electrolyte interfaces under realistic aqueous conditions, especially for water-splitting applications where the interplay between surface structure, hydration, and charge transfer governs catalytic efficiency.

Given the (not-negligible) higher cost associated with modeling fully explicit solid–liquid interfaces at the DFT-MD level, it is indeed critical to explore and adopt methods that balance accuracy with tractability. A number of strategies -outlined below but not limited to these- can be employed to improve efficiency, such as:
i) Reduced Supercell Size with Constrained Dynamics: in our current setup, large simulation cells (876–984 atoms) were used to capture a statistically meaningful solvent slab with realistic water density. In future work, smaller or asymmetric cells can be employed with selective freezing of deeper layers of the catalyst (usually bottom layers) to reduce the number of atoms involved in MD integration without sacrificing surface fidelity. This approach could maintain interfacial realism while lowering cost.
ii) DFT-MD with Machine-Learned Potentials: recent progress in machine-learning interatomic potentials (MLIPs) such as Gaussian approximation potentials (GAP), moment tensor potentials (MTP), or deep neural networks (e.g., DeepMD) offers a promising alternative. These potentials are trained on DFT datasets and can reproduce DFT accuracy at orders-of-magnitude lower cost, enabling simulations of larger cells and longer timescales. Once trained, MLIPs can handle interfacial systems with explicit water, including dynamics, thermodynamics, and structural transitions, with accuracy.
iii) Hybrid Implicit-Explicit Solvent Models: hybrid schemes where a few layers of explicit water are modeled near the interface, while the bulk solvent is represented using an implicit continuum model, offer a compromise between cost and accuracy. This approach captures essential hydrogen bonding and dipole effects near the surface, while reducing the number

of explicit atoms required.
